# Ex Vivo Organoid Cultures Reveal the Importance of the Tumor Microenvironment for Maintenance of Colorectal Cancer Stem Cells

**DOI:** 10.3390/cancers12040923

**Published:** 2020-04-10

**Authors:** Xingru Li, Pär Larsson, Ingrid Ljuslinder, Daniel Öhlund, Robin Myte, Anna Löfgren-Burström, Carl Zingmark, Agnes Ling, Sofia Edin, Richard Palmqvist

**Affiliations:** 1 Department of Medical Biosciences, Pathology, Umeå University, 90185 Umeå, Sweden; xingru.li@umu.se (X.L.); par.larsson@umu.se (P.L.); anna.lofgren-burstrom@umu.se (A.L.-B.); carl.zingmark@umu.se (C.Z.); agnes.ling@umu.se (A.L.); sofia.edin@umu.se (S.E.); 2 Department of Radiation Sciences, Oncology, Umeå University, 90185 Umeå, Sweden; ingrid.ljuslinder@umu.se (I.L.); daniel.ohlund@umu.se (D.O.); robin.myte@umu.se (R.M.); 3 Wallenberg Center for Molecular Medicine, Umeå University, 90185 Umeå, Sweden

**Keywords:** organoid, colorectal cancer, cancer stem cell, molecular profiling, tumor microenvironment

## Abstract

Colorectal cancer (CRC) is a heterogeneous disease, with varying clinical presentations and patient prognosis. Different molecular subgroups of CRC should be treated differently and therefore, must be better characterized. Organoid culture has recently been suggested as a good model to reflect the heterogeneous nature of CRC. However, organoid cultures cannot be established from all CRC tumors. The study examines which CRC tumors are more likely to generate organoids and thus benefit from ex vivo organoid drug testing. Long-term organoid cultures from 22 out of 40 CRC tumor specimens were established. It was found that organoid cultures were more difficult to establish from tumors characterized as microsatellite instable (MSI), *BRAF*-mutated, poorly differentiated and/or of a mucinous type. This suggests that patients with such tumors are less likely to benefit from ex vivo organoid drug testing, but it may also suggest biological difference in tumor growth. RNA sequencing analysis of tumor sections revealed that the in vivo maintenance of these non-organoid-forming tumors depends on factors related to inflammation and pathogen exposure. Furthermore, using TCGA data we could show a trend towards a worse prognosis for patients with organoid-forming tumors, suggesting also clinical differences. Results suggest that organoids are more difficult to establish from tumors characterized as MSI, *BRAF*-mutated, poorly differentiated and/or of a mucinous type. We further suggest that the maintenance of cell growth of these tumors in vivo may be promoted by immune-related factors and other stromal components within the tumor microenvironment.

## 1. Introduction

Colorectal cancer (CRC) is the third most commonly diagnosed cancer and the second leading cause of cancer related death in the developed countries [1]. Despite that mortality has decreased over the past decades due to improved treatment strategies, the 5-year relative survival rate is around 60%. An increased understanding of the molecular background of tumors will lead to further improvements in the management of CRC patients. CRC is a heterogeneous disease that develops as the result of genetic and epigenetic changes that drive different pathways of tumor progression. CRC has been sub-grouped based on different molecular characteristics, i.e., chromosomal instability, microsatellite instability (MSI) and the CpG island methylator phenotype, with distinct histological and clinical differences [2,3,4,5]. Patients with different molecular subgroups of CRC would likely benefit from different treatment strategies and, therefore, must be better characterized. However, until today in vitro models have not adequately reflected the heterogeneous nature of CRC.

Self-renewal of the colonic epithelium is driven by stem cells located at the base of the crypts in the colonic epithelium [6]. The long-term organoid culture of human colonic cells had previously not been achievable until Sato et al. developed a long-term organoid culture system that allowed isolated colonic cells to maintain basic crypt physiology [7]. The culture system consists of a laminin-rich extracellular matrix (matrigel) conditioned with a combination of Wnt3A, Roof Plate-Specific Spondin-1 (R-spondin1), Noggin, and epidermal growth factor (EGF), together with a TGF-β inhibitor (A83-01) and a p38 MAP Kinase inhibitor (SB202190). R-spondin1 is a ligand for Lgr5, which is essential to activate WNT signaling in intestinal crypts. EGF is associated with intestinal proliferation and Noggin induces increased crypt numbers. Activated WNT signaling is required to maintain active crypt stem cells in normal colonic epithelium. However, CRC cells are less dependent on WNT factors [7,8]. Therefore, the ex vivo growth of CRC cells requires no additional Wnt3A or R-spondin1 [8,9,10].

Based on this culture system, routine organoid cultures derived from both tumors and paired healthy epithelium from CRC patients have been established in several studies [8,10,11,12]. Established organoids efficiently form, self-renew, differentiate, self-organize, and expand. Unlike monolayer cell cultures that do not represent the functional and genetic heterogeneity of human cancers, organoid cultures closely recapitulate several properties of the original tumor with all major subtypes of CRC represented [10,11].

However, organoid cultures have not been established from all CRC tissues [7,13]. It has been described that different factors can impact the establishment of organoid cultures. Tissues derived from rare CRC histological subtypes (e.g., poorly differentiated adenocarcinoma, mucinous adenocarcinoma, and neuroendocrine carcinoma), advanced stage colon cancers and rectal cancers were less likely to form organoids [8]. Additionally, growth factors such as R-spondin1 and EGF, as well as a p38 inhibitor are believed to promote proliferation of some organoids while inhibiting growth of others [8].

The successful rate of establishing CRC organoids varies among different studies from 67.5% to 81% under standard culture conditions [8,10,12]. Fujii et al. have optimized the standard organoid culture protocol and observed that out of 40 established CRC organoids, three required WNT activators, five favored hypoxia and nine exhibited significant growth reduction with SB202190 (a p38 inhibitor) treatment. Collectively, 32.5% of the established CRC organoids were unable to grow under conditions with EGF, Noggin, A83-01, and SB202190 at atmospheric oxygen concentrations [8]. Moreover, eight combinatorial culture conditions were routinely applied for all the CRC cells isolated from different patients to establish organoids with 100% efficiency. This step is costly and time consuming and, therefore, not easy to apply to routinely establish organoid cultures.

The present study examines which CRC patients are more likely to generate organoid cultures, and thus would benefit from ex vivo drug testing. First, we adopted the previously published organoid-establishing method and generated long-term organoid cultures from 22 out of 40 CRC tumors. The cultures presented a range of patient-specific morphologies and well represented the morphologies and genetic landscape (i.e., *KRAS* and *BRAF* mutations and MSI status) of primary tumor specimens. By studying primary tumor specimens and comparing organoid-forming and non-organoid-forming tumors, we found that organoid cultures were more difficult to establish from tumors characterized as MSI, *BRAF*-mutated, poorly differentiated, and/or of a mucinous type. This suggests that patients with such tumors are less likely to benefit from ex vivo organoid drug testing, but it may also suggest biological differences in tumor growth. Using RNA sequencing analysis, we further explored the biological and clinical differences between organoid-forming and non-organoid-forming tumors. We found that the maintenance of the non-organoid-forming tumors in vivo was partly dependent on factors produced by immune cells within the tumor microenvironment, possibly in response to inflammation and pathogen exposure.

## 2. Results

### 2.1. Establishment and Characterization of Long-Term CRC Organoid Cultures

We were able to generate long-term organoid cultures from 22 out of 40 CRC tumor samples. Among the 22 established CRC organoid cultures, 16 were derived from colon tumors and six from rectal tumors. No growth was observed for 17 organoid cultures. One of the organoid cultures was discontinued due to bacterial infection. In total, we observed a 55% success rate. 

**Figure 1:**
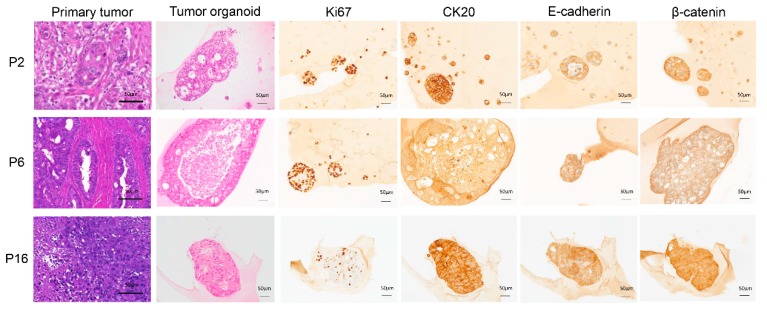
Organoid architecture resembles that of the primary tumor epithelium. Examples of H&E staining of tumor tissue and the paired tumor-derived organoid culture from three of the 22 established organoid cultures. IHC staining for different tumor markers was performed on FFPE organoids, as indicated: Ki67, proliferation marker; CK20, pan differentiation marker; E-cadherin, epithelial marker; β-catenin, intracellular signal transducer in the WNT signaling pathway. Magnification: 20×, scale bar: 50 µm.

The clinicopathological characteristics of the CRC patients are shown in Table S1. The established organoid cultures were derived from CRC tumors of different grades and stages, suggesting that they well represent the primary tumors. The growth rate varied between organoids derived from different patients, as exemplified in Figure S1. All the established CRC organoids could be expanded and maintained under the same conditions for at least one month and, underwent at least five passages, and were frozen down to create an organoid biobank. H&E staining of the established organoid cultures was compared with that of the corresponding primary tumor tissues and revealed patient-specific morphologies (Figure 1). Representative organoid samples were further IHC stained for different tumor markers (Figure 1).

MSI status, *KRAS,* and *BRAF* mutation status were also characterized and compared between primary tumor tissues and tumor-derived organoids from 15 patients (Figure S2). Seven samples were not analyzed due to an insufficient amount of DNA. Two of the primary tumors (P21 and P34) were identified as MSI. However, only one was maintained in an organoid culture (P34). *KRAS* mutations were observed in five primary tumors (P18, P19, P20, P24, and P39) and paired tumor-derived organoids. However, one tumor with a wild-type *BRAF* (P16) was identified with a *BRAF* mutation in the tumor-derived organoid culture. Another two patients (P33 and P34) were identified as carrying a *BRAF* mutation in paired primary tumors and tumor-derived organoids. The observations showed that the organoid cultures, to a large extent, captured the morphological and genomic features of the corresponding primary tumor.

### 2.2. Establishment of Organoid Cultures in Relation to Clinicopathological Characteristics and Molecular Subtypes

**Figure 2:**
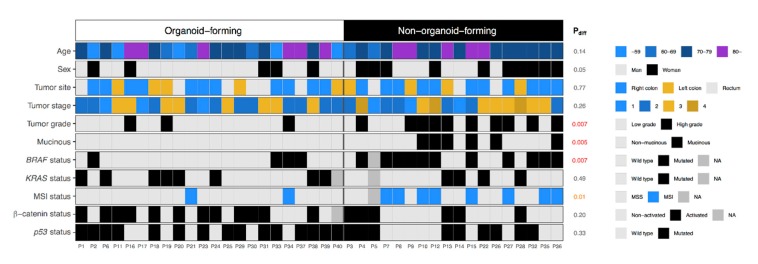
Establishment of organoid cultures in relation to the clinicopathological characteristics of their matching primary tumors. Comparison of the molecular background of organoid-forming tumors and non-organoid-forming tumors.

We studied the establishment of organoid cultures in relation to patient clinical and pathological characteristics to understand the difference between organoid-forming tumors and non-organoid-forming tumors (Figure 2). Findings showed clear molecular differences between the two groups (Figure 2). Compared with organoid-forming tumors, more non-organoid-forming tumors were characterized as MSI (*p* = 0.01), carrying a *BRAF* mutation (*p* = 0.007), poorly differentiated (*p* = 0.007), and were of the mucinous type (*p* = 0.005). Organoid cultures from female patients were more difficult to establish (*p* = 0.05, Figure 2). However, this result is not statistically significant and could be explained by the fact that *BRAF*-mutated CRCs are more common in female patients clinically and are poorly differentiated [14].

### 2.3. Comparison of the Organoid-Forming and Non-Organoid-Forming Tumors Using RNA Sequencing Analysis

**Figure 3:**
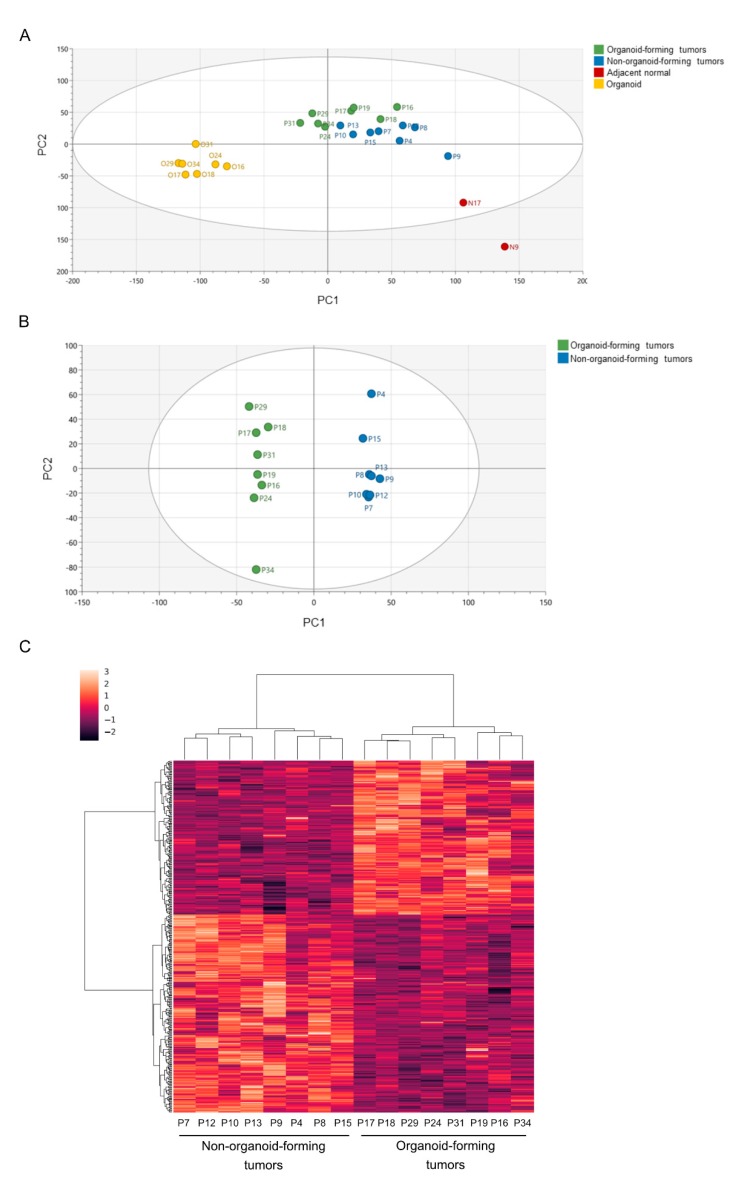
RNA sequencing analysis using primary tumors, organoids and tumor adjacent normal tissues. (**A**) OPLS-DA model of sequenced samples represent score scatter plots distinguishing organoid-forming tumors and non-organoid-forming tumors (P), adjacent normal tissues (N), and organoid samples (O); PCA plot of organoid-forming tumors and non-organoid-forming tumors (**B**) based on differentially-expressed genes between the two groups, as shown in a heat map (**C**).

To further study the molecular differences between organoid-forming and non-organoid-forming tumors, we analyzed the whole genome expression of 16 CRC tumor specimens in our cohort, of which eight were from organoid-forming tumors and eight were from non-organoid-forming tumors (Figure 3A) using RNA sequencing. We also included two samples of tumor-adjacent normal tissue and seven organoid samples (Figure 3A). One organoid sample was excluded from the RNA sequencing analysis due to low DNA input. An OPLS-DA model was established using the SIMCA software, and three classes (primary tumors, organoids, and tumor-adjacent normal tissues) were defined for the model (Figure 3A) [15]. By analyzing the 16 primary tumor samples closer, the OPLS-DA model could clearly separate the organoid-forming and non-organoid-forming tumors into two distinct groups (Figure 3B) based on differences in gene expression as illustrated in a heatmap (Figure 3C).

We identified 453 differentially-expressed genes between the two groups (111 genes with enriched expression in organoid-forming tumors and 342 genes with enriched expression in non-organoid-forming tumors) using a criterion based on fold change and *p*-value (Log2 fold change of 1 and *p* < 0.05) (Table S2). Among the differentially expressed genes, we found several genes involved in the regulation of stem cell maintenance and the immune and inflammatory response (Table S2).

Of the 111 enriched genes in organoid-forming tumors, four genes were found to be involved in stem cell proliferation. LGR6 (leucine rich repeat containing G protein-coupled receptor 6) has been identified as a marker of multipotent stem cells in the epidermis and is associated with phosphorylated LRP6 and frizzled receptors that are activated by extracellular WNT receptors, triggering the canonical WNT signaling pathway [16,17,18,19]. LGR6 is homologous to LGR5, which marks small intestinal stem cells at the crypt base [16]. Another enriched gene was *IGF2BP1* (insulin like growth factor 2 mRNA binding protein 1), which is crucial for colonic mucosal wound healing [20]. IGF2BP1 can also bind to the 3-UTR of CD44 mRNA and stabilize it, hence promoting cell adhesion [21]. CD44 has been suggested as a CRC stem cell marker [22]. RNF43 (ring finger protein 43) acts in both the canonical and non-canonical WNT signaling pathway [22]. TRIM71 (tripartite motif containing 71) maintains the growth and upkeep of embryonic stem cells [23]. Of the 342 enriched genes in non-organoid-forming tumors, we found 28 genes that were related to the immune response (for example: *CCL1*, *LYZ*, *CD109*, *CXCL6*, *CXCL8*, and *TLR1*); four genes were involved in inhibiting β-catenin-dependent WNT signaling (*DKK1*, *DCDC2*, *ROR2*, *FZD2*) [24,25,26,27]; and two genes were involved in the TGF-β signaling pathway (*BMPR1B* and *TGFBR3L*).

By comparing organoid-forming tumors and non-organoid-forming tumors, Gene Ontology (GO) enrichment analysis revealed that stem cell proliferation was among the most enriched Gene Ontology terms in organoid-forming tumors, while complement activation, the Fc-gamma receptor signaling pathway involved in phagocytosis, the regulation of immune response and inflammatory response were significantly enriched in non-organoid-forming tumors (Table 1). These results indicate that WNT signaling is not as essential for tumor growth in the in vivo setting of non-organoid-forming tumors, but instead growth may partly be driven by stromal factors produced in the tumor microenvironment, such as immune and inflammatory factors.

**Figure 4:**
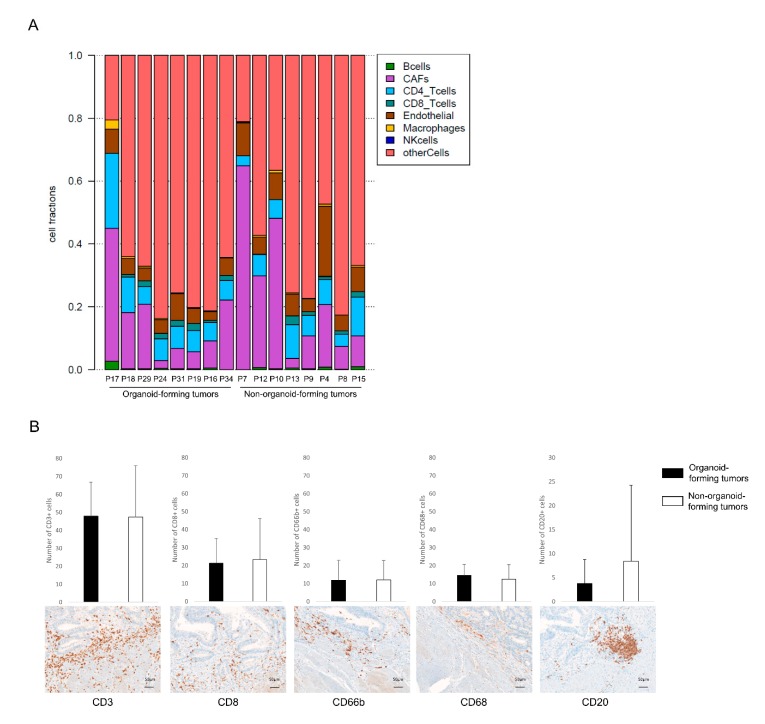
Identification of different immune cell subpopulations in the colorectal cancer (CRC) tumor microenvironment. Estimate of the Proportion of Immune and Cancer cells (EPIC) from tumor RNA sequencing data (**A**); the number of positively stained immune cells between organoid-forming and non-organoid-forming tumors and examples of positive staining for each marker (**B**).

In light of this finding, we used the Estimate of the Proportion of Immune and Cancer cells (EPIC) software to estimate the proportion of various immune cell types, as well as stromal cells, in the different tumor samples (Figure 4A). However, no significant differences were found between organoid-forming and non-organoid-forming tumors. The infiltration of different immune cell subpopulations; T cells (CD3), cytotoxic T cells (CD8), neutrophils (CD66b), macrophages (CD68), and B cells (CD20), was further evaluated at the tumor front using IHC staining, and the two groups were compared (Figure 4B). No significant difference was found in the number of infiltrating immune cells. This finding implies that it is not the subsets of immune cell or the number of immune cells, but rather their functional orientation (i.e., the inflammatory factors they produce) that accounts for this difference.

Two hundred and eighty-nine genes were found differentially-expressed between organoid cultures and their corresponding primary tumors, of which the main part (259 genes) were enriched in primary tumors (Table S3). Functional and pathway analysis revealed that the main differences could be attributed to the tumor microenvironment, including both immune- and inflammatory-response related pathways, which are not present in the organoid cultures (Table S4). Only one pathway (tRNA aminoacylation for protein translation) was upregulated in the organoid cultures. The results suggest that ex vivo organoid culture well preserved the functions of the primary tumor. However, in vivo tumor growth is likely stimulated by the microenvironment, in particular by immune and inflammatory components, even though not essential for self-sufficient growth in this setting.

### 2.4. Survival Analysis of Organoid Establishment Status in the Cancer Genome Atlas (TCGA) Database

**Figure 5:**
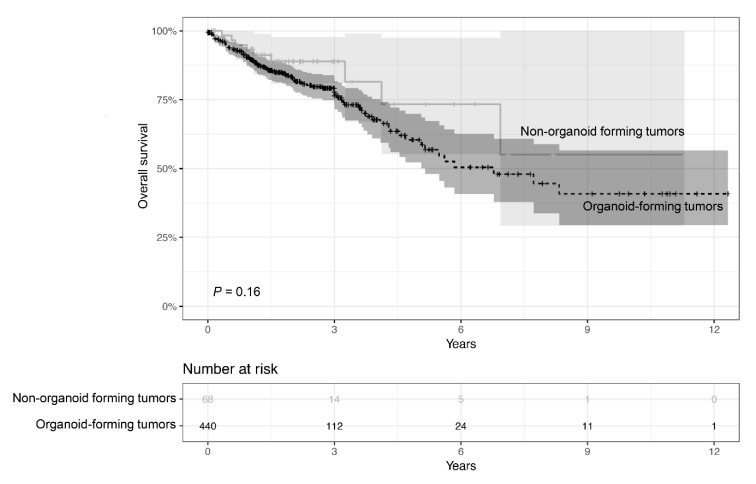
Kaplan–Meier survival analysis of patients according to organoid establishment status in the TCGA database. The overall survival of patients with organoid-forming versus non-organoid-forming tumors is shown.

To study the prognostic value of tumor organoid establishing ability, RNA sequencing data of 513 patients diagnosed with CRC was obtained from the TCGA database. Patients in the TCGA database were grouped into two groups based on the 453 differentially-expressed genes between the organoid-forming and non-organoid-forming tumors found in our cohort. Kaplan–Meier survival analysis was then performed, and the CRC patients with organoid-forming tumors (n = 440) showed a trend towards worse overall survival than those with non-organoid-forming tumors (n = 68; *p* = 0.16, Figure 5). 

## 3. Discussion

The present study generated long-term organoid cultures from 22 out of 40 CRC tumors. The organoid cultures well represented the morphologies and genetic landscape (i.e., *KRAS* and *BRAF* mutations and MSI status) of the primary tumor specimens. IHC analysis of the tumor-derived organoids presented a range of patient-specific morphologies. More importantly, we found that it was difficult to establish organoid cultures from tumors characterized as MSI, *BRAF*-mutated, poorly differentiated, or of a mucinous type. This suggests that patients with tumors of these molecular subtypes are, as of today, less likely to be candidates for ex vivo drug testing under the standard organoid culture condition. Future improvements in organoid establishment may overcome this challenge. On the molecular level, RNA sequencing analysis further revealed that the in vivo maintenance of non-organoid-forming tumors may be dependent on factors produced by immune cells in the tumor microenvironment. Using TCGA data we could further show a trend towards a worse prognosis for patients with organoid-forming tumors.

The success rate of generating CRC organoids was 55% in our study, which was lower compared to other studies ranging from 67.5% to 81% under standard culture conditions [8,10,12]. The slightly lower success rate may be partly explained by a higher number of MSI tumors in our cohort (25%) compared to the literature (10–15%) [28,29,30]. 

We demonstrated that the organoid cultures represented the most common molecular changes of CRC primary tumors, including *KRAS* and *BRAF* mutations, and the MSI phenotype. The frequency of *KRAS*-mutated organoid cultures was five out of 15 in our cohort (33.3%). *BRAF* mutation was found in three of 15 organoids (20%). MSI was identified only in one organoid line (6.7%). Discordant molecular characteristics were found in two patients. One patient was identified with MSI in the primary tumor but not in the corresponding organoid culture. This may be explained by the growth of one sub-clonal population in the organoid culture that either was not present at all within the original tumor, or was present within a different part of the tumor due to tumor heterogeneity. Another patient was found to have a *BRAF* mutation in the organoid culture but not in the primary tumor. This could be explained by tumor heterogeneity or may be due to the acquisition of additional mutations during organoid culture. It is known that patients with tumors of different molecular subtypes display varying prognoses and respond differently to treatment. In modern colorectal cancer treatment, chemotherapy is often combined with anti-EGFR antibodies to improve response rate. It is well known that the presence of *KRAS* and *BRAF* mutations in a patient’s tumor correlates strongly with resistance to anti-EGFR antibodies, and therefore, all patients are tested for *BRAF* and *KRAS* mutations before initiating anti-EGFR therapy [31]. In an unselected population, approximately 10% of patients respond to the therapy. Nevertheless, when treating *KRAS*/*BRAF*-wild-type patients exclusively, only 20–30% benefit from the anti-EGFR antibody [32]. It is, therefore, important to evaluate the efficacy of current therapies in relation to the molecular subtype of CRC. Organoid cultures could be a potential model for the design of such a personalized treatment plan.

The study also examined the establishment of organoid cultures in relation to the clinical and pathological characteristics of their matching primary tumors and found that it was more difficult to establish organoid cultures from tumors characterized as MSI, *BRAF*-mutated, poorly differentiated, or of a mucinous type. Whether or not organoid cultures can be established from MSI CRC tumors was not clear. Todaro et al. found that MSI and high-grade tumors form spheroids more efficiently and have suggested that MSI colon carcinomas might contain specific mutations that facilitate their in vitro growth [33]. Functional studies, however, have shown that genomic instability in epithelial cells activate the apoptotic program, and these cells were consequently eliminated from the tissue. In mammals, this was found to be dependent on p53 activity [34]. Sadanandam et al. have demonstrated a clear association between MSI and an inflammatory-subtype and between microsatellite stable and stem-like subtypes in CRC [35,36]. It has also been shown repeatedly that MSI-high CRCs were associated with high-level immune infiltrates [37]. Even though we were able to establish organoid cultures from some *BRAF^V600E^*-mutated tumors, most of those tumors did not form organoids. Contrary to our results, Kang et al. have found in mice that organoids carrying the *BRAF^V600E^* mutation were able to form xenograft tumors that exhibit the histological characteristics of human mucinous adenocarcinoma [38]. Those authors also found that *BRAF^V600E^* organoids are associated with increased expression levels of WNT pathway target genes, indicating an enhanced and sustained WNT signaling.

Survival analysis using TCGA data showed that there is a trend towards a better prognosis for patients with tumors that cannot form organoids. Previous studies have shown that *BRAF* mutations are more often found in MSI tumors [39,40]. Samowitz et al. have reported that MSI was associated with a good prognosis in primary CRC [41]. By combining MSI and *BRAF* mutational status, Seppälä et al. have demonstrated that *BRAF*-mutated MSI tumors were associated with a better prognosis than *BRAF*-mutated MSS CRC tumors [42]. Li et al. have found that *BRAF*-mutated CRCs were more frequently associated with infiltrating lymphocytes, high grade and mucinous appearance [40]. Ogino et al. have showed that *BRAF* mutations and MSI were more frequent among mucinous tumors than non-mucinous carcinoma [43]. All the above described previously published findings were based on primary CRC tumor tissues. The clinicopathological data in relation to the tumor’s organoid-forming ability and patient survival appear to be consistent. Our findings indicate that patients with tumors that could more easily form organoids had a worse prognosis, and thus would benefit from ex vivo organoid drug testing.

RNA sequencing results showed that of the significantly upregulated genes in organoid-forming tumors, several were involved in the WNT-signaling pathway. In contrast, WNT inhibitors were found enriched in non-organoid-forming tumors. These findings indicate that WNT signaling is important in maintaining tumor-initiating cells ex vivo. As demonstrated in previous studies, WNT signaling drives stem-cell proliferation in several mammalian tissues [44]. The self-renewal ability of the tissue was lost when the WNT pathway is inhibited [45]. In addition, the activation of WNT signaling has been found in most CRC cases [46].

Functional analysis revealed that the inflammatory response was significantly enriched in non-organoid-forming tumors. This finding may indicate that an inflammatory environment could promote in vivo CRC tumor growth. Previous studies have demonstrated that inflammatory processes are associated with CRC tumorigenesis [47,48,49], and that the microbiota has the potential to shape the inflammatory microenvironment in the intestine [50]. 

Several of the significantly enriched genes in non-organoid-forming tumors are immunoglobulins. Functional analysis showed that those genes are significantly associated with complement activation and the Fc-gamma receptor signaling pathway, involved in phagocytosis, the regulation of immune response, and inflammatory response. Previous studies have shown that patients can develop antibodies against tumor-associated antigens (TAAs) [51], and TAAs have been found to be associated with a poor-prognosis in cancer [52]. Moreover, increased levels of immunoglobulins in the tumor microenvironment could lead to the accumulation of immune complexes that activate tumor-promoting inflammatory responses [53,54]. Immunoglobulins could also activate the complement system. Complement activation in the tumor microenvironment is an important component of tumor-promoting inflammation [55]. Clinical data shows complement activation in CRC patients [56] and Ytting et al. have found that MASP-2, a protease responsible for activating the complement cascade, has an independent prognostic value in CRC patients, and that a high level of MASP-2 significantly correlates with recurrent cancer [57].

We compared proportions of various immune cell types between organoid-forming and non-organoid-forming tumors based on RNA sequencing data and IHC staining of tissues, and found no significant differences. Our findings may indicate an overexpression of CD20^+^ B cells in non-organoid-forming tumors, however the result was not statistically significant. Due to the small sample size in each subgroup (*n* = 8), final conclusions are hard to draw. However, the finding may imply that it is not the immune cell subsets or the number of immune cells, but rather their functional orientation and the different cytokines they secrete or present in the tumor microenvironment that account for this difference. For example, CD68^+^ macrophages could be of both M1 and M2 type with different cytokine profiles and functional impact on tumor progression [58]. Similarly, CD8^+^ T cytotoxic cells may be either activated or inhibited depending on cytokines in the tumor microenvironment [59]. Further analyses of functional subgroups of immune cells are required to fully understand the role of the immune response in maintenance of CRC stem cells.

Due to the limited number of collected samples available for analyses in this study, all the findings need to be verified in larger cohort.

## 4. Materials and Methods

### 4.1. Study Population of Patients with CRC

Patients included in the study were from the Uppsala-Umeå Comprehensive Cancer Consortium (U-CAN) project [60]. Tissue specimens were collected from patients who had been surgically resected for CRC at the Department of Surgery, Umeå University Hospital, Sweden since 2010. Tumor tissues for organoid culture were sequentially derived from 40 out of 145 U-CAN patients from November 2015 until April 2017. All patients that had undergone pre-operative treatment were excluded. Other exclusion criteria included limited tumor size, surgeries out of laboratory working hours, lack of U-CAN referral to the pathology department, and other logistic problems. Both fresh frozen tissues and formalin-fixed paraffin-embedded (FFPE) tissues were sampled from the patients, along with clinical and pathological data. All subjects gave their written informed consent for inclusion before they participated in the study. The study was conducted in accordance with the Declaration of Helsinki. The handling of tissue samples and patient data was approved by the Regional Ethical Review Board of Umeå, Sweden (Approval number: 2016/218-31). The overview of the study population as well as the experimental analyses used in this study has been summarized in Figure S3.

### 4.2. Tumor Cell Isolation, Organoid Culture, and Storage

Fresh tumor tissue samples (5–10 mm) were collected at the time of routine sampling at the Department of Pathology. Tissue specimens were cut into small pieces in a petri dish using a clean scalpel. The Tumor Dissociation Kit and the Octo Dissociator (Miltenyi Biotec, Bergisch Gladbach, Germany) were used for tumor tissue dissociation and the isolation of tumor cells following the manufacturer’s instructions. In brief, tissue was inserted into a gentleMACS C Tube with a volume of 5 mL enzyme mix. The enzyme mix was consisted of 200 µL of Enzyme H, 50 µL of Enzyme R, and 12.5 µL of Enzyme A, and RPMI1640 cell culture medium. The C tube was then placed onto the gentleMACS Octo Dissociator with Heater and the program 37C_h_TDK1 was run. After tumor tissue dissociation, isolated tumor cells were mixed with matrigel (Corning, NewYork, NY, USA) and plated in basal culture medium (advanced Dulbecco’s modified Eagle medium/F12 supplemented with penicillin/streptomycin (ThermoFisher, Waltham, MA, USA), 10 mM HEPES and Glutamax) with the addition of 1× B27 (ThermoFisher, NewYork, NY USA), 1 mM n-Acetyl Cysteine (Sigma Aldrich, St Louis, MO, USA), 50 ng/mL human EGF (ThermoFisher, MD, USA), 10 nM Gastrin (Sigma Aldrich, St Louis, MO, USA), 500 nM A83-01 (Tocris, Minneapolis, MN, USA), 3 µM SB202190 (Sigma Aldrich, St Louis, MO, USA), and 10 µM Y-27632 (Sigma Aldrich, St Louis, MO, USA) and incubated at 37 °C with 5% CO_2_. The organoid culture medium was changed every two days and organoids were passaged every week. Matrigel was scraped off from the bottom using a pipette to passage the organoids. Organoids were then broken into single cells by pipetting up and down 10–15 rounds and collected in a tube. After centrifugation at 350× g for five minutes at 4 °C, the supernatant was aspirated, and the pellet was mixed with matrigel and plated in 24 well plates. The organoids were stored in liquid nitrogen in a basal culture medium containing 10% DMSO for long-term storage.

### 4.3. Immunohistochemistry

Tumor tissue specimens and organoids were fixed in 4% formaldehyde and embedded in paraffin. FFPE blocks were cut into 4-μm sections, dried, de-waxed and rehydrated. The sections were subjected to H&E staining and immunohistochemical (IHC) staining using an automated Ventana Benchmark Ultra staining machine with the ultraVIEW DAB Detection Kit for visualization (Ventana Medical Systems, Inc., Tucson, AZ, USA). The following antibodies and dilutions were used for IHC staining: anti-Ki-67 (Mouse monoclonal, Clone MIB-1, Catalogue No. M7240, DAKO, Carpinteria, CA, USA), 1:50; anti-CK20 (Mouse monoclonal, Clone Ks20.8, Catalogue No. M7019, DAKO, Carpinteria, CA, USA), 1:200; anti-E-cadherin (Rabbit polyclonal, Catalogue No. SC-7870, Santa Cruz, CA, USA), 1:100; anti-β-catenin (Rabbit polyclonal, Catalogue No. C2206, Sigma, St. Louis, MO, USA), 1:750; anti-p53 (Mouse monoclonal, Catalogue No. NCL-L-p53-DO7, Leica, Buffalo Grove, IL, USA), 1:100; and five immune markers were used for tumor tissue specimens: anti-CD3 (Rabbit monoclonal, Clone 2GV6, Catalogue No. 790-4341, Ventana, Tucson, AZ, USA); anti-CD8 (Mouse monoclonal, Catalogue C8/144B, Catalog No. M7103, DAKO, Carpinteria, CA, USA), 1:50; anti-CD66b (Mouse monoclonal, Clone G10F5, Catalogue No. 555723, BD Pharmingen, San Jose, CA), 1:400; anti-CD68 (Mouse monoclonal, Clone KP1, Catalogue No. M0814, DAKO, Carpinteria, CA, USA), 1:800; and anti-CD20 (Mouse monoclonal, Clone L26, Catalogue No. ab9475, abcam, Cambridge, UK), 1:50. The stainings were evaluated with light microscopy by one observer under the supervision of an experienced gastropathologist. 

β-catenin was assessed for intensity and percentage of cells with positive nuclear staining, as previously described [61]. Briefly, the intensity was scored as 0–3 (0 = no staining; 1 = weak staining; 2 = moderate staining; 3 = strong staining), whereas percentage was scored as 0–4 (0 = no staining; 1 = 1–10%; 2 = 11–50%; 3 = 51–90%; 4 = ≥90% of cells). An activated β-catenin status required nuclear staining with a score of ≥2 in the intensity or percentage category. When the *APC* gene is inactivated, accumulated β-catenin translocates from the cell membrane to the nucleus, where it drives the transcription of multiple genes implicated in tumor growth and invasion [62]. Therefore, β-catenin nuclear expression was assessed using IHC.

The p53 IHC staining was assessed as previously described [63,64]. IHC patterns of wild type p53 is characterized by variable proportions of tumor cell nuclei showing a mixture of negative, weak and strong p53 staining. Aberrant p53 IHC patterns are defined as: Overexpression (OE), cytoplasmic (CY), or complete absence (CA). OE is the most common pattern and is characterized by strong nuclear staining of most tumor cells. The CY pattern is characterized by diffuse cytoplasmic staining with lack of strong nuclear staining. CA is characterized by absence of nuclear staining of tumor cells but detectable nuclear staining of p53 in non-malignant stromal cells such as fibroblasts and lymphocytes [63,64].

IHC staining of the five immune markers was assessed at five randomly chosen areas of the tumor invasive tumor front. The tumor front was identified as the stromal area along the invasive margin defined by a depth of one high-power field (×40 objective magnification) underneath the invasive margin. Areas of necrosis were avoided. The number of positively stained cells was counted. The specimens were evaluated twice by the same observer and discordant cases were reviewed a third time, followed by a conclusive judgment.

### 4.4. DNA and RNA Extraction

DNA and RNA were extracted from five 10 µm FFPE sections of tumor and corresponding normal tissues using the AllPrep DNA/RNA FFPE kit (Qiagen, Sollentuna, Sweden). The AllPrep DNA/RNA/miRNA Universal kit (Qiagen, Sollentuna, Sweden) was used for fresh frozen tumor tissues, corresponding adjacent normal tissues and organoid cultures, following the manufacturer’s instructions. Prior to extraction, a 2–3 mm cube of fresh frozen tissues was homogenized using the Precellys 24 homogenizer (Bertin Technologies, Rockville, Washington, DC, USA) with 1.4 mm ceramic beads (Soft tissue homogenizing CK14 in a 0.5 ml tube, Bertin Technologies, Rockville, Washington, DC, USA). The quantity of extracted DNA and RNA was assessed using the Qubit dsDNA BR Assay Kit and the Qubit RNA BR Assay Kit (Invitrogen, Carlsbad, CA, USA), respectively. The quality was evaluated using TapeStation 2200 (Agilent Technologies, CA, USA).

### 4.5. Microsatellite Instability (MSI) Assessment

MSI analysis was performed with the MSI Analysis System Version 1.2 (Promega, Madison, WI, USA) consisting of mononucleotide repeats *BAT-25*, *BAT-26*, *NR-21*, *NR-24*, *MONO-27* (and pentanucleotide repeats *Penta C* and *Penta D* for sample identification) [65]. To detect MSI, 10 ng DNA from each sample was amplified with 1× primer mix, 1× Gold STAR Buffer (Promega, Madison, WI, USA) and AmpliTaq Gold® DNA Polymerase (Applied Biosystems, Thermo Fisher Scientific, Forster City, CA, USA) in a Veriti® 96-Well Thermal Cycler (Applied Biosystems, Thermo Fisher Scientific, Forster City, CA, USA) following the manufacturer’s recommended amplification conditions for the MSI Analysis System. PCR products were denatured in deionized formamide with Internal Lane Standard 600 (Promega, Madison, WI, USA) for allele sizing and analyzed on a 3130*xl* Genetic Analyzer using GeneMapper 4.0 Software (Applied Biosystems, Forster City, CA, USA). Allelic profiles of matching tumor and normal samples were compared. The appearance of novel alleles in the tumor samples in a comparison with their matched normal samples indicates MSI according to the manufacturer’s instructions.

### 4.6. BRAF^V600E^ Mutational Status

Digital droplet PCR (ddPCR, Bio-Rad Laboratories, Hercules, CA, USA) was used to detect the *BRAF^V600E^* mutation. The ddPCR method is presented thoroughly elsewhere [66,67]. Briefly, a PCR sample of 20 µl was partitioned into 20 000 nanolitre droplets. The PCR reaction was then performed in a T100 Thermal Cycler (Bio-Rad Laboratories, Hercules, CA, USA) using the program: 95 °C for 10 min; 40x cycles of 95 °C for 15 s; and 56 °C for 1 min (ramp rate 2 °C/s); and 98 °C for 10 min. 900 nM of the primers and 250 nM of each probe were used. The primers and probes for *BRAF^V600E^* mutation detection were as follows: forward: 5′-GCACAGGGCATGGATTACTTACA-3′, reverse: 5′-ATCCAGACAACTGTTCAAACTGATG-3′, wild type probe: 5′-56-FAM/TTGGTCTAGCTACAGTGAAAT/3BHQ_1-3′, mutation probe: 5′-5HEX/TTGGTCTAGCTACAGAGAAAT/3BHQ_1-3′ (DNA Technology A/S, DNA Technology A/S) [68].

### 4.7. KRAS Sequencing

Mutations of *KRAS* gene have been sequenced at the codon12 or 13 on exon 2. The experiment details were explained by Eklöf V et al. [39]. In brief, the Sanger sequencing was carried out on a 3730*xl* DNA Analyser using Big Dye v. 3.1 (Applied Biosystems, Life Technologies, Stockholm, Sweden) and the primers used were: forward: 5’-TGTAAAACGACGGCCAGTGAGTTTGTATTAAAAGGTACTGG-3’ and reverse: 5’-CAGGAAACAGCTATGACCTCTGTATCAAAGAATGGTCCT-3’.

### 4.8. RNA Sequencing and Analysis

A total amount of 1 μg RNA per sample was used as input material for the RNA sequencing reactions.

The RNA sequencing library construction was performed using NEBNext Ultra RNA Library Prep Kit for Illumina (NEB, USA) following the manufacturer’s recommendations. Index codes were added to attribute sequences to each sample. The clustering of the index-coded samples was performed on a cBot Cluster Generation System using HiSeq PE Cluster Kit cBot-HS (Illumina, CA, USA) according to the manufacturer’s instructions. After cluster generation, the library preparations were sequenced on an Illumina Hiseq platform and 125 bp/150 bp paired-end reads were generated. 

Trimmed fastq files were mapped to the human genome version GRCH38 using HISAT2 to generate alignment files [69,70]. The DESeq2’s rlog function was used to generate the PCA plot. An orthogonal projection to latent structures discriminant analysis (OPLS-DA) model was established using SIMCA v.16.0.2 (Umetrics/Sartorious-Stedim, Sweden) and classifications of the samples were defined for the model [15]. OPLS-DA is a supervised classification method that models categorical factors (organoid-forming and non-organoid-forming classes) as a function explanatory quantitative variables X (transcripts) and is useful when explanatory variables highly multidimensional and collinear. The data was scaled by centering only. The validity of the model was assessed using R2, Q2, permutation tests and pCV-ANOVA. Salmon (v0.8.2, https://combine-lab.github.io/salmon/) was used to quantify the expression of transcripts [71,72].

Differential gene expression between organoid-forming tumors and non-organoid-forming tumors; and between organoid cultures and their corresponding primary tumors was analyzed using the DESeq2 package in R [73]. Sex and batch were used for normalization. For analyzing the significantly differentially expressed genes, multiple testing was corrected for using false discovery rate as a part of the results generated by Deseq2 function in R. Significantly differentially expressed genes were filtered for *p*-value < 0.05 and log2 fold change set as ≥ 2 or ≤ −2.

Functional annotation of the differentially expressed genes was performed using the Database for Annotation, Visualization and Integrated Discovery (DAVID) [74,75]. Analyses of enriched biological processes and pathways were annotated using the Gene Ontology Consortium (http://www.geneontology.org/) and the Kyoto Encyclopedia of Genes and Genomes database (KEGG, https://www.genome.jp/kegg/).

An EPIC analysis from bulk tumor gene expression data was performed as previously described [76]. EPIC is based on a unique collection of RNA-Seq reference gene expression profiles from either circulating immune cells or tumor- infiltrating non-malignant cell types (i.e., immune, stromal and endothelial cells).

RNA sequencing data was also obtained from the TCGA database (COAD and READ), and 513 CRC patients with survival data were analyzed. Data was normalized as described above. A PCA analysis was performed for the TCGA dataset based on the identified differentially expressed genes in our CRC cohort. The patients from the TCGA data set were then classified into groups with either organoid-forming or non-organoid-forming tumors based on the PCA analysis.

### 4.9. Statistical Analysis

Statistical analysis was performed using PASW Statistics 25 (SPSS Inc., Chicago, IL, USA).

Cross-tabulations for associations between categorical variables were analyzed with the *χ*^2^ test. The non-parametric Kruskal–Wallis H and Mann–Whitney U tests were used to compare differences in continuous variables between groups. Fisher’s exact test was used when the variable count was less than five. The Kaplan–Meier survival analysis was used to estimate overall survival and the log-rank test was used to compare the differences in outcomes between two patient groups. Overall survival was defined as from the date of diagnosis to death of any cause. All statistical analyses were two-sided and *p*-values ≤ 0.05 were considered statistically significant.

## 5. Conclusions

We successfully generated long-term organoid cultures from 22 out of 40 CRC tumors. By comparing the matching tumor derived organoids and primary tumors, we found that the organoids well represented the morphologies and genetic landscape (*KRAS*, *BRAF,* mutation and MSI) of the corresponding primary tumor specimens. We also found that it was more difficult to establish organoid cultures from tumors characterized as MSI, *BRAF*-mutated, poorly differentiated, or of a mucinous type. RNA sequencing analysis further revealed that the in vivo maintenance of non-organoid-forming tumors may be dependent on factors produced by immune cells in the tumor microenvironment, possibly in response to inflammation and pathogen exposure.

## Figures and Tables

**Table 1:** Significantly enriched Gene Ontology (GO) terms in non-organoid-forming tumors.

Annotated Functions	*p*-Value	Number of Genes
Complement activation	<0.001	19
Complement activation, classical pathway	<0.001	19
Fc-gamma receptor signaling pathway involved in phagocytosis	<0.001	18
Receptor-mediated endocytosis	<0.001	20
Regulation of immune response	<0.001	19
Immune response	<0.001	25
Fc-epsilon receptor signaling pathway	<0.001	17
Proteolysis	<0.001	24
O-glycan processing	0.006	7
Maintenance of gastrointestinal epithelium	0.027	4
Inflammatory response	0.029	14

## Supplementary Materials

The following are available online at https://www.mdpi.com/2072-6694/12/4/923/s1, Figure S1. The time course for establishment of representative organoid cultures derived from three patients with CRC. Light microscopic images of organoid cultures taken at day 1, 3, 7, and 14 after plating with a magnification of 20× (Scale bar: 50 µm), Figure S2. Molecular characterization of primary tumors and corresponding tumor organoids. Analyses shown include microsatellite instability (MSI) status, and *KRAS* and *BRAF* mutation, Figure S3. Overview of the study cohort and the experimental analyses performed in the present study, Table S1. Clinicopathological characteristics of CRC patients in this study, Table S2. Differentially expressed genes between organoid-forming and non-organoid-forming tumors, Table S3. Differentially expressed genes between organoid cultures and their corresponding primary tumors, Table S4. Significantly enriched GO terms in organoid-forming tumors compared to organoids.
