# Supplementary material for: Ex Vivo Organoid Cultures Reveal the Importance of the Tumor Microenvironment for Maintenance of Colorectal Cancer Stem Cells
Source: Cancers (Basel). 2020 Apr 10;12(4):923. doi: 10.3390/cancers12040923 (PMC7226030; doi:10.3390/cancers12040923)
Supplement: Supplementary file 1 [file cancers-12-00923-s001.pdf]

cancers

Supplementary Materials: Ex Vivo Organoid Cultures Reveal the Importance of the Tumor Microenvironment for Maintenance of Colorectal Cancer Stem Cells

Xingru Li, Pär Larsson, Ingrid Ljuslinder, Daniel Öhlund, Robin Myte, Anna Löfgren-Burström , Carl Zingmark, Agnes Ling, Sofia Edin, Richard Palmqvist

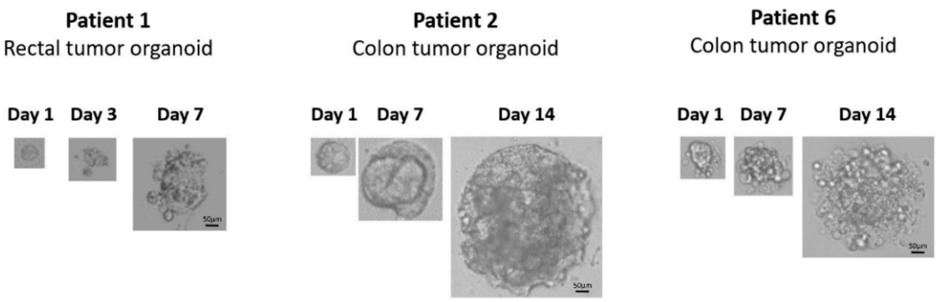

Figure S1. The time course for establishment of representative organoid cultures derived from three patients with CRC. Light microscopic images of organoid cultures taken at day 1, 3, 7, and 14 after plating with a magnification of 20× (Scale bar: 50 µm).

Primary tumor

	MSI*	*KRAS*	*BRAF*
**P11**	MSS†	WT‡	WT
**P16**	MSS	WT	WT
**P17**	MSS	WT	WT
**P18**	MSS	G12V	WT
**P19**	MSS	G12V	WT
**P20**	MSS	G12D	WT
**P21**	MSI	WT	WT
**P24**	MSS	G13D	WT
**P25**	MSS	WT	WT
**P29**	MSS	WT	WT
**P30**	MSS	WT	WT
**P31**	MSS	WT	WT
**P33**	MSS	WT	V600E
**P34**	MSI	WT	V600E
**P39**	MSS	G12V	WT

Tumor organoid

	MSI	*KRAS*	*BRAF*
**P11**	MSS	WT	WT
**P16**	MSS	WT	V600E
**P17**	MSS	WT	WT
**P18**	MSS	G12V	WT
**P19**	MSS	G12V	WT
**P20**	MSS	G12D	WT
**P21**	MSS	WT	WT
**P24**	MSS	G13D	WT
**P25**	MSS	WT	WT
**P29**	MSS	WT	WT
**P30**	MSS	WT	WT
**P31**	MSS	WT	WT
**P33**	MSS	WT	V600E
**P34**	MSI	WT	V600E
**P39**	MSS	G12V	WT

*MSI, microsatellite instability; †MSS, microsatellite stable; ‡WT, wild type.

Figure S2. Molecular characterization of primary tumors and corresponding tumor organoids. Analyses shown include microsatellite instability (MSI) status, and KRAS and BRAF mutation.

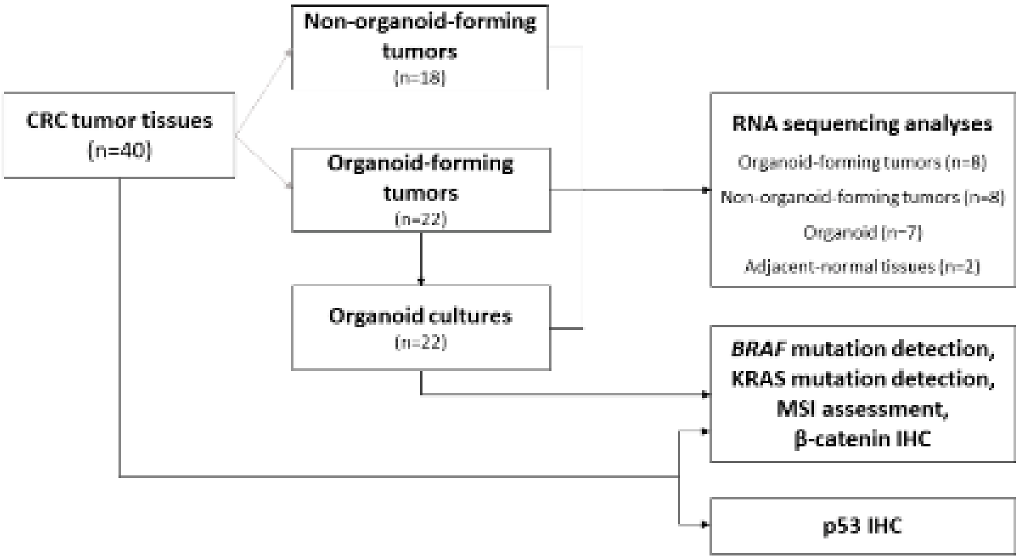

Figure S3. Overview of the study cohort and the experimental analyses performed in the present study.

Table S1. Clinicopathological characteristics of CRC patients in this study.

	**Colorectal**		**Colon**		**Rectum**	
**Location**	**Total**	**Established**	**Total**	**Established**	**Total**	**Established**
	n	*n* (%)	*n*	*n* (%)	*n*	*n* (%)
**Sex**						
**Male**	25	17 (68)	18	12 (66.7)	7	5 (71.4)
**Female**	15	5 (33.3)	13	4 (30.8)	2	1 (50)
**Age**						
**≤ 59 years**	5	5 (100)	4	4 (100)	1	1 (100)
**60-69 years**	5	3 (60)	3	2 (66.7)	2	1 (50)
**70-79 years**	19	8 (42.1)	14	5 (35.7)	5	3 (60)
**≥ 80 years**	11	6 (54.5)	10	5 (50)	1	1 (100)
**Grade**						
**Low (highly differentiated)**	27	19 (70.4)	19	13 (68.4)	8	6 (75)
**High (low differentiated)**	13	3 (23.1)	12	3 (25)	1	0
**Stage**						
**I**	6	3 (50)	4	3 (75)	2	0 (0)
**II**	17	11 (64.7)	14	10 (71,4)	3	3 (100)
**III**	14	8 (57.1)	10	6 (60)	4	3 (75)
**IV**	3	0	3	0	0	0 (-)

Table S2. Differentially expressed genes between organoid forming and non-organoid forming tumors.

Title	**log2FoldChange**	***p*-Value**	**adjusted *P*-value**
SFTA3	25.12687725	1.48E-11	6.06E-08
LINC02128	22.51161922	7.29E-10	1.97E-06
AL365217.1	20.21123763	5.83E-08	7.20E-05
AC026355.2	20.18426538	5.85E-08	7.20E-05
FO393401.2	19.96868625	8.39E-08	9.67E-05
AC063952.2	19.88192911	9.08E-08	0.000101555
NKX2-1-AS1	19.81908282	1.04E-07	0.000112625
AC020913.1	19.71484281	1.23E-07	0.000122206
AC126696.1	19.60730084	1.43E-07	0.000135543
KRT18P39	19.49105389	1.70E-07	0.000152903
EIF4A1P2	19.40092159	1.94E-07	0.000170258
TRAJ37	18.92997307	3.82E-07	0.000320288

LINC01539	18.8150687	4.50E-07	0.000361024
AF241726.2	18.68596405	5.40E-07	0.000377812
AC012313.7	18.34143894	8.73E-07	0.000519687
AL132857.1	18.17063518	1.08E-06	0.000585527
AL109935.2	17.55768874	2.19E-06	0.001051034
AC116616.1	17.50357946	2.64E-06	0.001204375
LCN9	17.28625933	3.55E-06	0.001411383
AC016629.1	17.20345826	8.35E-10	2.05E-06
ZNF840P	17.11317906	4.46E-06	0.001679252
GJA10	16.72949875	7.77E-06	0.002725814
SLC6A2	16.71530192	7.91E-06	0.002725814
NEAT1_3	16.67649674	7.85E-06	0.002725814
SNX18P7	16.63427966	8.28E-06	0.002727145
AC008740.2	16.63427952	8.28E-06	0.002727145
GVINP2	16.63427943	8.28E-06	0.002727145
INE2	16.63427937	8.28E-06	0.002727145
MIR4252	16.49782382	9.92E-06	0.002951767
AP007216.1	16.49782361	9.92E-06	0.002951767
AC092436.3	16.49782359	9.92E-06	0.002951767
AL773604.1	16.49782359	9.92E-06	0.002951767
AP000553.5	16.49782359	9.92E-06	0.002951767
LY6G6D	9.144217962	2.48E-07	0.00021264
LY6G6F-LY6G6D	7.64292128	4.40E-05	0.008423616
NKX2-1	6.961764065	3.64E-09	7.90E-06
GNG4	6.826612709	1.25E-12	6.57E-09
SLC22A11	6.167076259	5.43E-07	0.000377812
FREM2	6.046469997	4.86E-07	0.000372827
ZNF663P	5.981868914	0.000472691	0.040457317
RBP2	5.734484937	2.29E-06	0.001068745
CEL	5.159536848	1.65E-06	0.000835058
AL662899.1	5.059559944	0.000401359	0.036320337
UGT2B10	4.974165493	1.41E-05	0.003943936
AC147055.1	4.874413839	0.000167239	0.021026404
CYP4F23P	4.708373231	3.37E-05	0.007308318
KRT23	4.685704187	4.85E-06	0.0017884
AL031686.1	4.659843495	0.000479891	0.040787944
MAP7D2	4.654360973	0.000457265	0.039503614
CRAT37	4.451313715	0.000599646	0.047983383
MYH4	4.33623522	0.000591774	0.04766064
ACE2	4.237550916	0.000114493	0.015877949
F7	4.232502776	3.23E-06	0.001323044
CASC21	4.065275456	0.000608058	0.048049115
TRIM71	3.989894529	3.78E-05	0.007916862
AP003774.4	3.950696631	3.94E-06	0.001547289
MGC32805	3.942628146	2.78E-05	0.00651295
PTPRO	3.93099947	3.41E-05	0.007366826
CELP	3.873123912	0.000590354	0.047653357
VENTX	3.868188061	7.01E-06	0.002510451
DNAH17-AS1	3.820736186	2.29E-05	0.005553933
LINC01555	3.808982552	0.000123945	0.016859163
SATB1-AS1	3.724987235	8.01E-05	0.012414855
MSI1	3.723172969	0.000169715	0.021168918
DUSP9	3.673852156	0.000363053	0.03477417
SHISA9	3.59460031	9.44E-07	0.000529405
POU5F1B	3.554731905	0.000245507	0.026953884
BCL11A	3.527669633	0.000171582	0.021168918
GJC2	3.504029864	5.60E-05	0.009656075
GRM8	3.469468998	8.22E-05	0.012530091
LGR6	3.462767723	0.000151231	0.019438187
FOLR1	3.46007266	5.41E-05	0.009462572
AC011447.7	3.368342113	0.000510905	0.042639743
PIPOX	3.260090501	0.000107859	0.01536217
HUNK	3.21760687	5.70E-05	0.009656075
PKHD1	3.137452621	5.64E-05	0.009656075

KCNH3	3.103702079	2.00E-05	0.00513145
IGF2BP1	3.023474422	3.68E-05	0.007759721
CDH26	2.946346286	4.82E-05	0.008853983
IL33	2.680733542	0.000400871	0.036320337
CKB	2.680472344	0.000257898	0.027736405
ST6GAL1	2.515012182	4.20E-05	0.008283024
ABAT	2.498121932	0.000290055	0.029875392
AL161772.1	2.424183787	3.52E-05	0.007506967
RNF43	2.233641807	5.18E-05	0.009206436
ZNF423	2.226996508	0.000588171	0.047648147
ASGR1	2.183947353	0.000167577	0.021026404
ARID3A	2.169667344	0.000394722	0.036320337
SP6	2.160725666	1.86E-05	0.004841556
GRK3	2.052523184	3.50E-05	0.00750105
GGH	2.010435729	4.86E-05	0.008878403
SLC5A6	1.915712917	0.000139141	0.018331391
DHRS2	1.691714664	0.000567869	0.046640779
CBX2	1.676870314	0.000403569	0.036320337
PABPC1L	1.489383771	4.95E-05	0.008950558
RNFT2	1.487008267	0.00060455	0.048049115
IGF2BP2	1.438568433	7.71E-05	0.012101757
SCML1	1.368484023	0.000144351	0.018950086
ASXL1	1.311707516	0.000480897	0.040787944
PFDN4	1.306514848	0.000211398	0.024678098
AC004656.1	1.253160785	0.00056896	0.046640779
AC106886.2	1.241446302	0.000604213	0.048049115
DLEU2	1.220073623	0.000418075	0.037073013
MSH6	1.217034025	5.66E-05	0.009656075
TFDP2	1.193396731	4.69E-05	0.008789878
MSH2	1.165505673	7.46E-05	0.011803927
MBIP	1.157896584	0.000493193	0.041547589
HDAC2	1.073937674	0.000108333	0.015370311
HIC2	1.068274382	0.000282022	0.029305637
TSC22D2	1.064176918	4.61E-05	0.00870724
IQCB1	1.034590915	7.93E-05	0.012336057
LRP10	-1.038597552	0.000626791	0.04926934
NLRX1	-1.126614696	0.000166472	0.021026404
FAM109B	-1.129031926	0.000218083	0.024946469
MTMR9LP	-1.178131702	5.51E-05	0.009580435
TBC1D2	-1.230491355	4.12E-05	0.008283024
IFIH1	-1.230498469	0.000198077	0.023723627
FBXO10	-1.335623434	0.000233116	0.025901879
TSPAN3	-1.375119254	0.000107486	0.01536217
RASSF6	-1.390840119	4.74E-05	0.008836737
LINC00893	-1.405228533	0.000570685	0.046678451
LIX1L	-1.453297766	0.000437515	0.038245282
APOL4	-1.508604455	0.000227505	0.025518941
THEMIS2	-1.543382208	0.000261817	0.02799472
DTX1	-1.559606739	1.86E-05	0.004841556
TCEAL3	-1.580097503	0.000382655	0.036009566
AKAP3	-1.617779365	0.000410952	0.036529199
STARD9	-1.61808547	0.00019941	0.023778908
AC010615.2	-1.619414417	0.000531351	0.044146408
AC022364.1	-1.627217013	0.000232133	0.0258706
GABARAPL1	-1.628026755	0.000250231	0.027229411
PTAFR	-1.632578613	0.000584753	0.047513075
RNF207	-1.633153439	0.000182073	0.022240058
TM6SF1	-1.637549767	0.000320162	0.031920154
GPR162	-1.640957114	2.13E-05	0.005366989
SLC15A3	-1.662940901	7.26E-05	0.011598742
CYP2U1	-1.705132204	0.00040487	0.036320337
ADORA2A	-1.714692777	0.000390379	0.036273767
GSN	-1.732268585	9.27E-06	0.002901006
KLF9	-1.759666542	0.000635944	0.049701963

ANKRD34A	-1.785787612	1.83E-05	0.004841556
GLIDR	-1.839037559	0.000148191	0.01924113
AC012146.1	-1.849610113	0.000310195	0.031161198
RPS6KA2	-1.865835503	0.000452318	0.039352757
FRMD3	-1.922144148	0.000499974	0.041917168
PDE4B	-1.922835043	2.28E-05	0.005553933
OASL	-1.97563589	0.000279752	0.029194112
STYK1	-1.985859037	0.000265378	0.02829339
NR4A2	-1.987486107	0.000398396	0.036320337
C15orf59	-2.01426866	0.000642826	0.049922547
TPBG	-2.027620566	2.47E-05	0.005923551
PTGFR	-2.039156722	0.000480977	0.040787944
TTC28	-2.039472022	1.84E-05	0.004841556
ARHGAP22	-2.060310559	1.63E-05	0.004448786
FZD2	-2.064900376	0.000306354	0.030961932
MIR22HG	-2.068845613	0.000180838	0.0221626
OAS2	-2.073543477	0.000588998	0.047648147
SYTL4	-2.079027864	2.09E-08	3.21E-05
LINC00239	-2.091615978	4.90E-05	0.008899924
SCNN1A	-2.10071044	0.000520821	0.043369259
DNAH7	-2.111854845	0.000581273	0.047334648
TFAP2A-AS1	-2.116943309	0.000608283	0.048049115
LANCL3	-2.123347495	0.000229026	0.025601604
ELOVL7	-2.147944182	6.27E-05	0.010326351
GSEC	-2.149124145	5.22E-05	0.009206436
MUC1	-2.149204883	2.79E-06	0.001255447
SETBP1	-2.167511234	4.17E-05	0.008283024
ALOX5	-2.19359485	4.78E-05	0.008853983
CFAP73	-2.198965749	0.00048223	0.040800455
GMPR	-2.229839664	0.000150555	0.019418906
GNE	-2.233213065	0.000641794	0.049922547
AP002004.1	-2.235841198	0.000112683	0.015685917
CHADL	-2.259829829	0.000233945	0.02591596
B3GALNT1	-2.291757422	0.000461052	0.039552865
AL031847.1	-2.298221801	0.000454515	0.039358218
EPB41L4A	-2.300440683	0.000110023	0.015459849
TLR1	-2.313700397	0.000405102	0.036320337
SAMD9L	-2.331754146	0.000218385	0.024946469
COLEC11	-2.339266108	0.000394118	0.036320337
AC025171.5	-2.348499439	0.000397978	0.036320337
LPAR1	-2.351896935	0.000497727	0.041823786
IFI6	-2.377844162	2.78E-05	0.00651295
PRKAR2B	-2.387697941	0.000121433	0.016652604
HIVEP3	-2.428951496	0.000148655	0.01924113
COL28A1	-2.436779726	2.34E-05	0.005632876
AL583722.1	-2.438035283	0.00031086	0.031161198
AC005077.4	-2.488486129	0.00024526	0.026953884
C1orf132	-2.492612451	6.24E-07	0.00041867
ZNF300	-2.495598498	0.000460917	0.039552865
TRABD2B	-2.517459387	5.28E-07	0.000377812
SSPN	-2.523173019	4.36E-05	0.008420039
SPTB	-2.551459992	0.000396028	0.036320337
VSIG1	-2.551881366	0.000137918	0.018235336
SCD5	-2.558487409	4.44E-05	0.008441176
MT1L	-2.57070405	0.000504454	0.04219687
ICAM5	-2.592120553	0.000303322	0.030824335
PTGER2	-2.608322067	4.41E-05	0.008423616
LINC02320	-2.661767588	7.82E-05	0.012227783
ADAM28	-2.662678745	9.34E-05	0.013617869
SAMD9	-2.663431697	8.20E-05	0.012530091
AGPAT4	-2.677834682	2.17E-05	0.005366989
FKBP1B	-2.679098462	0.000278931	0.029194112
SLC16A14	-2.708195118	0.000131053	0.017579622
SLC24A3	-2.720463022	0.000404375	0.036320337

ZNF304	-2.736758955	5.71E-05	0.009656075
FAM149A	-2.748855153	6.98E-05	0.011290062
GCNT3	-2.763201498	2.65E-05	0.006317759
SIX4	-2.769169854	0.000257579	0.027736405
ZG16B	-2.773426864	5.65E-05	0.009656075
ZDBF2	-2.807724741	2.89E-06	0.001269987
ARHGAP6	-2.810645945	5.88E-06	0.002125786
RIMS3	-2.821954568	1.79E-05	0.004796067
LAMA1	-2.846488734	0.000364811	0.03477417
MUC4	-2.860049955	0.000280157	0.029194112
LINC01422	-2.860539452	0.000363908	0.03477417
MTCL1	-2.86788145	0.000112482	0.015685917
ENTPD8	-2.873688988	0.000594321	0.04766064
CHRNA7	-2.885608768	0.000208466	0.02449073
PCDHGB2	-2.887545388	5.98E-05	0.009980802
ADAMDEC1	-2.899007862	0.000351406	0.034203245
C14orf37	-2.900297904	7.21E-07	0.000443377
CXCL8	-2.920629658	0.000422647	0.037210108
EIF4E3	-2.931395607	1.62E-09	3.73E-06
PLCL2	-2.935753939	6.50E-07	0.000428177
VNN2	-2.979083441	0.00025785	0.027736405
HPD	-2.998498029	0.00035692	0.034377119
MGAM	-3.002351163	6.46E-05	0.010593795
DPYD	-3.020410792	1.53E-05	0.004246581
AP002498.1	-3.021423935	0.00024026	0.026535832
ABCG5	-3.068005373	0.000102385	0.014753448
ROR2	-3.087566498	0.000201683	0.023830643
LRRN2	-3.090184904	0.000410087	0.036529199
BHLHA15	-3.152609126	0.00021885	0.024946469
GFI1	-3.162221237	2.60E-06	0.001197798
CXCL6	-3.175108144	0.000137194	0.018204824
TLL2	-3.179643601	0.000154283	0.019761658
AL121761.2	-3.190858522	2.28E-05	0.005553933
AC114488.2	-3.197624677	0.000627204	0.04926934
LUCAT1	-3.200141043	0.000128845	0.017346591
SLCO4A1-AS1	-3.200804983	0.000266291	0.028308997
PRDM8	-3.251965316	0.000302389	0.030814449
GALNT8	-3.260559907	0.000218884	0.024946469
ST6GALNAC5	-3.264876068	3.56E-06	0.001411383
ST3GAL4	-3.265427456	4.60E-09	8.93E-06
LTK	-3.291758507	3.14E-06	0.001323044
PRTG	-3.309515742	0.000159991	0.020421838
CHRFAM7A	-3.325465287	0.000225139	0.025465244
B3GNT7	-3.354006659	8.17E-05	0.012530091
SECTM1	-3.375421945	8.46E-05	0.012790175
SNCA	-3.402857594	0.00011684	0.01608244
TGFBR3L	-3.428971023	0.000373643	0.035251408
KCND3	-3.429455312	0.000538134	0.044509502
SDK2	-3.444186152	0.000196833	0.023651334
CHST6	-3.458292758	2.26E-08	3.34E-05
SPDEF	-3.476927657	0.000184333	0.022294569
GRIP2	-3.497364675	0.000544647	0.044896764
SNPH	-3.520580752	0.000110221	0.015459849
KCNJ3	-3.525569747	5.77E-05	0.009706301
CD109	-3.561982246	4.07E-08	5.72E-05
LGI2	-3.591874393	7.25E-05	0.011598742
PLXNB3	-3.61356492	0.000279531	0.029194112
ADRB1	-3.630791824	4.68E-06	0.001743369
FFAR4	-3.634770419	6.71E-07	0.000434244
ISL1	-3.64243643	8.87E-06	0.002845369
ALDOB	-3.670541528	8.07E-06	0.002727145
ARHGEF4	-3.722464688	0.000183282	0.022250175
KCNQ4	-3.750999571	6.63E-13	4.07E-09
IGLC2	-3.766118614	0.000202201	0.023830643

LAIR2	-3.80809861	0.000387998	0.036200183
LINC02489	-3.809098422	0.000368026	0.034810572
IGLV1-47	-3.841801114	0.000593247	0.04766064
IGLV1-51	-3.869364814	0.000116759	0.01608244
SERPINA3	-3.870131955	2.16E-05	0.005366989
TNFRSF6B	-3.879511731	1.68E-05	0.004562589
AC009163.2	-3.887178614	0.000259056	0.027780003
POU2AF1	-3.907067179	6.92E-07	0.000440278
MYRFL	-3.923823505	0.000248761	0.027149571
SLFN13	-3.944939919	0.0001658	0.021026404
HOTAIR	-3.976588569	9.00E-05	0.013331866
IGKV1-17	-3.996314639	0.000185622	0.022377208
B3GALT5	-4.022393543	4.26E-05	0.008283024
AC022182.2	-4.025852069	0.000398363	0.036320337
FER1L6	-4.13193434	8.25E-05	0.012530091
AC003965.2	-4.152862529	4.27E-05	0.008283024
COLCA1	-4.197860322	0.000366439	0.034793986
RAMP1	-4.212941317	3.22E-06	0.001323044
FAM30A	-4.227308251	0.000460572	0.039552865
CHST5	-4.237422899	4.99E-07	0.000372827
MUC2	-4.255805475	0.00013425	0.017878454
CHRM1	-4.265436988	6.52E-05	0.010638051
LYZ	-4.277035942	5.05E-07	0.000372827
AC098850.4	-4.277110887	5.15E-05	0.009206436
EYA2	-4.292489757	0.000356165	0.034377119
RASGEF1A	-4.306595244	2.00E-05	0.00513145
XXYLT1-AS2	-4.317639698	0.000126857	0.017141512
CCNB3	-4.334808035	1.03E-05	0.003044692
IGFALS	-4.364605578	0.000132846	0.017755663
AC005832.4	-4.40226393	0.0003534	0.034306741
IGKV3-15	-4.412596169	0.000290744	0.029875392
ALDH1L1	-4.420965088	0.00035567	0.034377119
IGHV1-18	-4.433307361	0.00017847	0.02194532
AL049794.1	-4.436333506	0.000388606	0.036200183
GPR158	-4.463400107	0.000284651	0.029495728
IGHV4-39	-4.470717325	4.06E-05	0.008270661
VNN1	-4.480092195	7.22E-05	0.011598742
IGHJ4	-4.483496258	8.42E-06	0.002749562
BMPR1B	-4.494492452	0.00025242	0.027386863
MEGF10	-4.531388099	0.00014648	0.019093626
IGKV1-5	-4.555995672	3.83E-05	0.007967552
IGHV5-51	-4.610654954	4.82E-05	0.008853983
MTUS2	-4.665764265	0.000276607	0.029153597
AQP5	-4.707145099	0.000225735	0.025465244
IGHV4-4	-4.76006054	0.000545251	0.044896764
IGKV1-9	-4.771915029	0.000300461	0.030702817
IGHV3-21	-4.796614904	2.95E-05	0.006723533
IGLV2-11	-4.834615327	7.52E-05	0.011849566
KLHDC7A	-4.887844588	9.81E-06	0.002951767
AC010478.1	-4.888198774	8.84E-05	0.013150959
TEX101	-4.930973359	0.000171096	0.021168918
IGHV3-66	-4.977800473	0.00043744	0.038245282
FAM177B	-4.987228217	8.67E-07	0.000519687
IGKV2-30	-5.000471004	1.72E-05	0.004634739
ECEL1P2	-5.146871244	0.000349919	0.034148536
IGHG3	-5.185331672	3.09E-06	0.001323044
IGHV3-33	-5.199492429	9.11E-09	1.68E-05
AGBL4	-5.201609957	3.17E-05	0.006999354
DCDC2	-5.270299507	4.43E-06	0.001679252
IGHV3-11	-5.27518523	1.35E-05	0.003800307
IGHV4-61	-5.301352733	7.56E-06	0.002680346
IGKV1-27	-5.316330771	3.59E-05	0.007617531
LINC00261	-5.321660655	1.16E-06	0.000619341
LINC02475	-5.403790085	0.000219107	0.024946469

NBPF7	-5.435064768	8.60E-06	0.002782077
AC136616.2	-5.465221732	4.11E-05	0.008283024
IGLV7-43	-5.503379754	5.79E-05	0.009706301
IGLV2-23	-5.513933222	4.99E-07	0.000372827
IGHJ2	-5.5518925	0.000493314	0.041547589
IGHV1-69	-5.600858271	3.12E-05	0.006976497
CLDN18	-5.635733611	1.10E-05	0.003143723
IGHV1-3	-5.64738851	9.28E-06	0.002901006
IGHV4-28	-5.650857841	0.000607181	0.048049115
TCN1	-5.699884778	8.98E-07	0.00052269
IGKV1-39	-5.734401187	0.000171338	0.021168918
MAATS1	-5.74299057	8.14E-05	0.012530091
HRASLS	-5.755071449	0.000212892	0.024696151
IGLV2-14	-5.798379562	1.22E-07	0.000122206
IGHV4-31	-5.802636196	1.07E-05	0.003085294
IGLV3-9	-5.84874863	1.04E-05	0.003044692
IL17REL	-5.867928072	9.07E-07	0.00052269
IGLV4-69	-5.912041401	5.20E-05	0.009206436
IGKV1-6	-5.946481349	3.84E-05	0.007967552
IGLV10-54	-6.08778355	0.000607103	0.048049115
IGLV5-45	-6.182287738	4.27E-05	0.008283024
IGHJ5	-6.372471892	5.77E-06	0.002107515
IGHV3-19	-6.382217421	4.08E-06	0.001583984
IGHV3-43	-6.480388569	3.35E-05	0.007308318
IGKV2-24	-6.580042925	0.00064085	0.049922547
REG3A	-6.653732661	0.000210558	0.024657959
LINC00930	-6.696560513	0.000349657	0.034148536
IGHV3OR16-9	-6.76172094	4.63E-05	0.00870724
IGHV4-34	-6.789948447	1.08E-07	0.000113681
IGKV1D-8	-6.889180911	0.000439303	0.038310778
IGHV3-72	-7.020079928	0.000166224	0.021026404
DKK1	-7.124240663	5.32E-05	0.009345948
IGHV4-55	-7.547897488	3.09E-06	0.001323044
REG1A	-8.093548211	5.53E-07	0.000377812
REG1B	-8.142914956	0.000109569	0.015459849
AC022188.1	-13.29832979	0.000421135	0.037165628
AC010175.1	-13.30267671	0.000419305	0.037092933
AC022509.4	-13.3267388	0.000409311	0.036529199
AC087499.3	-13.33569483	0.000405649	0.036320337
AC006946.3	-13.38299576	0.000386715	0.036200183
AC245517.5	-13.38663388	0.000385398	0.036175457
AC096638.1	-13.43536084	0.000366908	0.034793986
LINC01800	-13.44102488	0.000364813	0.03477417
AC010894.4	-13.48883701	0.000347488	0.03409171
AC026725.1	-13.49074584	0.000346816	0.03409171
AC079584.2	-13.49436048	0.000345547	0.034082555
NTAN1P3	-13.53094741	0.000333025	0.03293558
TRAJ31	-13.55106288	0.000326197	0.032346976
MIR6891	-13.57874316	0.000317075	0.031698032
AL391903.2	-13.6068586	0.000308228	0.031066176
AC107871.2	-13.61828952	0.000304647	0.030873948
TRBV10-1	-13.66760389	0.000289567	0.029875392
AP003108.1	-13.71241672	0.000276414	0.029153597
AC020909.2	-13.82090264	0.00024693	0.027029715
IGKV6D-41	-13.90763601	0.000222999	0.025311449
KRT27	-13.95313712	0.000215411	0.024910056
AL132640.2	-13.98961921	0.000201663	0.023830643
TRDV2	-14.30404924	0.000146333	0.019093626
AC007216.1	-14.46146667	0.000124311	0.016859163
IGKV1-35	-14.55883873	1.35E-05	0.003800307
AC016747.2	-14.60800796	0.000107238	0.01536217
IGHV3OR16-16	-14.66122114	0.00010119	0.014696098
LINC00158	-14.66438179	1.02E-06	0.000562952
AC073055.1	-14.69999843	1.58E-05	0.004348429

RPE65	-14.72136851	2.89E-05	0.006620937
KIR2DS4	-14.73428202	7.48E-10	1.97E-06
GRM5-AS1	-14.74199181	1.68E-06	0.000839006
AC006205.2	-14.74299803	9.25E-05	0.013544212
AL691459.1	-14.75971506	9.08E-05	0.01334248
AC116903.1	-14.76557129	6.02E-05	0.010006139
CYCSP40	-14.79091678	8.78E-05	0.013109378
AC005920.2	-14.79268196	8.76E-05	0.013109378
CCL1	-14.8042547	1.60E-06	0.00081754
PDE6H	-14.80955087	8.60E-05	0.01294804
TRAV10	-14.82116934	4.18E-06	0.001604987
RN7SL482P	-14.93716534	7.45E-05	0.011803927
TRAV25	-14.94809996	1.06E-08	1.86E-05
TRBV11-1	-15.00180973	6.92E-05	0.01123933
IGLV4-3	-15.06481794	5.26E-08	6.93E-05
AC006116.1	-15.08201649	2.84E-06	0.001261417
RPL38P4	-15.09338804	6.19E-05	0.010233106
H3F3C	-15.0989142	3.18E-06	0.001323044
AC073370.1	-15.10255234	3.36E-06	0.001361959
HLA-DPA3	-15.20623391	1.61E-07	0.000148851
KRT72	-15.21469168	1.29E-06	0.00067772
CASC20	-15.23549135	5.13E-05	0.009206436
AC243962.1	-15.29923312	1.31E-07	0.000127633
AC017007.3	-15.39022138	4.16E-05	0.008283024
LINC02085	-15.39161733	4.21E-05	0.008283024
IGHV3-75	-15.39626158	4.02E-05	0.008241388
IGHV3OR16-15	-15.41349362	3.87E-09	7.92E-06
AL158210.1	-15.42000517	7.20E-07	0.000443377
AC009086.2	-15.42486758	3.96E-05	0.008152861
XKR3	-15.56651898	3.33E-05	0.007308318
AC022239.2	-15.60609579	1.48E-12	6.82E-09
AF192304.1	-15.61237245	3.14E-05	0.006984318
AC093899.2	-15.61751988	3.09E-05	0.006976497
LINC02205	-15.62800672	9.47E-07	0.000529405
SPRR2D	-15.66852558	2.79E-05	0.00651295
CSAG3	-15.67324314	2.83E-05	0.006567048
AC104662.2	-15.67462998	2.86E-05	0.00658948
AL031736.2	-15.74007076	4.06E-07	0.000333006
AC008063.1	-15.83567237	1.17E-10	3.92E-07
IGHV1-12	-15.87836021	2.16E-05	0.005366989
SLC32A1	-15.91005599	2.08E-05	0.005302106
LINC00582	-15.99547142	2.74E-13	2.02E-09
LRRC14B	-16.03767598	3.15E-10	9.68E-07
RN7SL339P	-16.2279508	9.69E-11	3.58E-07
PIK3CD-AS1	-16.41760543	1.23E-08	2.06E-05
IGLV3-22	-16.42219655	1.05E-05	0.00305314
AC007278.2	-16.45670569	1.39E-06	0.0007242
AC024681.2	-16.51594162	4.19E-08	5.72E-05
AC127496.6	-16.53711409	8.96E-06	0.00285044
TRDV1	-16.62687692	2.24E-06	0.00105846
NTSR2	-16.66170107	1.47E-08	2.35E-05
AC142381.1	-16.79703829	1.83E-06	0.000901195
AC099524.1	-16.93483807	1.39E-14	1.71E-10
AL121988.1	-16.99207048	6.52E-14	6.01E-10
IGHV1OR16-1	-17.65071403	1.65E-15	3.04E-11
BMS1P13	-18.82975706	6.08E-08	7.23E-05
IGKV6D-21	-19.66558542	1.39E-16	5.12E-12

Table S3. Differentially expressed genes between organoid cultures and their corresponding primary tumors.

Title	**log2FoldChange**	***p*-Value**	**adjusted *P*-value**
SMPX	17.22936479	5.92149E-07	0.000318335
AC243967.1	8.273478743	0.000625325	0.048028351
CREG2	8.105690493	0.000365534	0.033599793
KLK8	7.968443886	0.000248247	0.026427309
AC005186.1	7.365063349	8.0486E-06	0.002219474
GOLGA2P8	7.302647213	0.000441254	0.038695071
FGF19	7.266761038	0.000202784	0.022530724
UGT1A1	6.931958188	0.000124929	0.016307086
CYP1A1	6.722825394	0.000326598	0.030875307
ABCG2	6.711277142	0.000498492	0.041792973
AC009511.2	6.699048188	0.00016414	0.019471287
EVX1	6.216404936	0.000177049	0.020363344
SMARCA5-AS1	5.742405903	6.4542E-05	0.010118056
TMC1	5.435725051	0.000176204	0.020363344
LINC02320	4.811150209	6.05812E-05	0.009562642
HPD	4.512645662	0.000327801	0.030875307
AC007405.3	4.121233752	0.000318709	0.030875307
AC091959.3	3.993147336	2.07569E-05	0.004358571
MT-TY	3.850384541	0.000438038	0.038560783
AL445584.2	3.575664741	0.000554546	0.044534939
IGFL4	3.279794711	3.21068E-05	0.006219718
CHAC1	3.244080224	2.88917E-06	0.001017342
TPD52L1	3.164170524	7.73369E-05	0.011645305
TMEM144	2.843865004	0.000261884	0.027000022
LINC00239	2.437795021	0.000157778	0.019106965
ASNS	2.304656136	4.28074E-05	0.007422543
NQO1	1.957992914	0.000541896	0.044138467
TBX3	1.881069531	0.000167868	0.019805005
TMEM41B	1.818465764	4.38683E-05	0.007506741
CCNT1	1.643351071	2.72539E-06	0.001006107
AMN1	1.582821188	0.000599525	0.046992933
TMEM182	1.540629234	0.0005226	0.043026184
UNC119B	1.498162014	0.000153292	0.018796828
YARS	1.484446471	0.000575008	0.045382
IARS	1.42606645	3.50378E-05	0.006415567
DHX32	1.353191153	7.17238E-06	0.002060786
SAP130	1.198066637	0.000136221	0.01697737
XIAP	1.185882987	0.000232141	0.025064159
MARS	1.171420389	0.000312004	0.030780831
TBCD	-1.416537257	0.000469869	0.040669045
ELN	-2.528663651	2.9012E-05	0.005774155
NOTCH4	-2.646882192	0.000546487	0.044197835
FAM167B	-2.77316091	1.58885E-06	0.000637993
RN7SL2	-2.846847651	9.99304E-09	1.08915E-05
BMP8A	-2.870350491	0.000622465	0.048028351
ENTPD1	-2.888388989	0.000270569	0.027646316
GRASP	-2.902162497	0.000556655	0.04454801
43344	-3.007119436	0.000164189	0.019471287
43347	-3.085802828	0.000368865	0.033770343
SIGLEC10	-3.09961368	0.000230074	0.025064159
RASGRP4	-3.338437801	0.000133324	0.016952866
PVRIG	-3.40835252	0.000572976	0.045378077
PLEKHO1	-3.432668815	0.000322479	0.030875307
CSF1R	-3.538767608	0.000162158	0.019431801
TCF4	-3.542723627	0.000566232	0.045156545
PLXDC1	-3.545798708	4.17943E-06	0.001350733
CD37	-3.556524016	4.64198E-05	0.007755155
ARHGAP30	-3.59812828	0.00048306	0.040949168
IL2RA	-3.706237549	1.38615E-05	0.003270739
LAIR1	-3.733596203	0.000418357	0.037258159

FAM46C	-3.780680569	0.000407503	0.036576177
PREX1	-3.800528656	0.000175573	0.020363344
COL15A1	-3.826269318	0.000305371	0.030398887
ARHGAP9	-3.858336576	0.000274435	0.027916757
CLEC3B	-3.869022281	4.70486E-05	0.007803249
C16orf54	-3.879923392	0.000503566	0.042064318
SOX7	-3.908923124	0.000381775	0.0346749
PCED1B-AS1	-3.926242563	0.000257684	0.02668725
CD4	-3.94579056	0.00047903	0.040758495
NCF1	-3.959237429	0.000269419	0.027646316
IL21R	-3.960840014	0.000550185	0.044340235
NFATC2	-3.976130446	0.000192139	0.021748187
CCL22	-3.977453517	0.000322738	0.030875307
SCIMP	-4.011885383	0.00012014	0.01598706
CPNE5	-4.031229107	0.000125759	0.016307086
P2RY8	-4.060868599	7.31176E-05	0.011231653
ITGAL	-4.064816822	0.000410121	0.036667408
DPEP2	-4.088573494	1.45998E-05	0.003409799
KCNH3	-4.113127492	0.000173604	0.020362004
ECM2	-4.175028281	1.74167E-05	0.003946858
PCDH12	-4.195222439	1.14212E-05	0.002841385
DOCK8	-4.226345674	4.54942E-05	0.007665857
TM6SF1	-4.246268542	0.000531695	0.043618047
FCRL1	-4.266504825	0.000313983	0.030843084
EDNRA	-4.267222351	1.85414E-05	0.004080543
KCNQ3	-4.295394988	0.000326821	0.030875307
RASSF2	-4.303531123	8.4863E-05	0.012371613
SLCO2A1	-4.306694298	0.000123634	0.016262852
CYSLTR1	-4.324236827	3.49509E-05	0.006415567
FGL2	-4.324394365	0.000515138	0.042719128
CD180	-4.406330471	0.000256676	0.02668725
CYBB	-4.413742962	9.17666E-05	0.012940085
TAL1	-4.425060006	0.000344952	0.032225531
BTK	-4.44481975	0.000612097	0.047814621
HLA-DOA	-4.467094662	0.000488623	0.04126792
CD7	-4.504382232	0.000309271	0.030643304
FCGR1A	-4.508270715	0.000474479	0.040755893
AQP1	-4.522206688	2.99979E-07	0.000176049
CCR7	-4.523695812	0.000341407	0.032025069
IL7R	-4.534738652	0.000155277	0.018904196
POU2AF1	-4.609618211	1.64291E-05	0.003798264
TMEM204	-4.610769019	3.64736E-06	0.001209866
PTPRC	-4.616907762	0.000619234	0.048028351
ARL10	-4.637271887	7.22284E-05	0.011170022
SLA	-4.645290978	0.000144478	0.01787469
PTGIR	-4.651602812	0.000470871	0.040669045
TLR2	-4.67140644	0.00039395	0.035639222
HMCN1	-4.67675303	5.67294E-06	0.00170845
PTPN7	-4.712072658	2.05677E-05	0.004358571
ADAMTS4	-4.756372325	9.21546E-05	0.012940085
IGSF6	-4.788055986	2.07278E-05	0.004358571
CPXM1	-4.799216935	3.42144E-10	6.76296E-07
NOX4	-4.811724278	2.67529E-05	0.005371232
P2RY6	-4.818609826	7.7037E-05	0.011645305
KLHL6	-4.819681984	7.174E-07	0.000349939
PARVG	-4.829177425	0.000599225	0.046992933
ZNF423	-4.833711399	3.18391E-05	0.006219718
KLRB1	-4.838505472	0.000174369	0.020362004
CD209	-4.866069582	0.000153574	0.018796828
GIMAP7	-4.868611	5.76411E-05	0.009225804
SLAMF8	-4.878310672	2.33701E-05	0.004818869
LAPTM5	-4.885551166	3.38624E-06	0.001139769
APOE	-4.886978852	0.000361155	0.033331118
F2RL3	-4.899409268	8.64984E-06	0.002249744

CHST2	-4.904669196	2.64037E-05	0.005371232
APBB1IP	-4.929546471	8.97652E-05	0.012761154
CMAHP	-4.932398535	3.23378E-05	0.006219718
INMT	-4.949401816	0.000351529	0.032706478
ARHGAP15	-4.962588947	3.15682E-06	0.001094746
EVI2A	-4.999186869	2.6663E-05	0.005371232
LILRB4	-5.004961106	0.000535705	0.043790028
ARHGEF15	-5.04648282	9.03013E-06	0.002322265
AIF1	-5.086966252	9.4855E-05	0.013238057
NLGN4X	-5.122441173	0.000111043	0.015038733
TBC1D10C	-5.132320706	0.000134842	0.016957546
PIK3R6	-5.150701706	0.000134082	0.016955069
F13A1	-5.158952237	0.00013193	0.016869296
SPARCL1	-5.173968678	1.10141E-06	0.000475643
CHSY3	-5.184011596	0.000623832	0.048028351
CLEC7A	-5.221022949	1.86961E-06	0.000725284
GIMAP1	-5.275668086	1.76796E-07	0.000126453
SRGN	-5.284769517	0.000251688	0.026546662
CD53	-5.291163496	3.41865E-05	0.006413621
NCAM1	-5.294911957	2.89911E-07	0.000175932
SI	-5.325361907	0.000317572	0.030875307
MYO1G	-5.367581003	6.1749E-07	0.000321207
HLA-DQB2	-5.372412947	0.000401734	0.036200333
IGHG3	-5.3735264	0.000245677	0.026399313
CSF2RB	-5.384698293	8.13149E-05	0.012085289
TNFRSF4	-5.390177043	3.44746E-08	2.92242E-05
ST8SIA4	-5.401837918	9.96196E-05	0.013653249
MIR4458HG	-5.439859908	0.00019165	0.021748187
OLFML2B	-5.462190933	1.06697E-05	0.002713433
HLA-DQA1	-5.496805459	0.000282618	0.028370843
CD48	-5.504213577	3.21136E-06	0.001097038
GMFG	-5.514642939	8.03142E-10	1.41402E-06
HEYL	-5.515999632	4.96654E-09	6.31523E-06
CCDC180	-5.534855351	2.66946E-10	6.10985E-07
TPSB2	-5.545165111	0.000326708	0.030875307
KCNN3	-5.548921891	0.000115748	0.01558377
EDNRB	-5.556137459	7.50543E-07	0.00035058
IGHV4-59	-5.557275699	4.91656E-06	0.001520678
GPR4	-5.560671212	6.4019E-07	0.000325615
CXorf36	-5.56093196	4.41921E-12	3.37157E-08
GIPC3	-5.568278591	0.000127317	0.016371024
AF127577.1	-5.575485168	0.000232157	0.025064159
CD52	-5.577162558	4.0712E-05	0.007120485
COL11A1	-5.58915032	0.000475438	0.040755893
HCLS1	-5.624906441	1.97284E-05	0.004300423
CSF3R	-5.683971785	7.99323E-06	0.002219474
P2RX1	-5.707659401	4.39489E-05	0.007506741
DOK2	-5.72629778	5.11797E-05	0.008367144
AC051649.2	-5.789125634	0.000434106	0.038362221
VWF	-5.800365884	3.41772E-11	1.86987E-07
MGP	-5.830655959	2.47881E-09	3.33736E-06
TRBC2	-5.855098755	5.55696E-05	0.009020398
MATK	-5.858315489	0.000136484	0.01697737
FAM19A5	-5.860349512	3.54577E-10	6.76296E-07
CD69	-5.877404553	0.000276893	0.02804211
AP000879.1	-5.878461065	9.6478E-05	0.013302342
CLDN5	-5.899721932	7.46772E-07	0.00035058
THBD	-5.901799319	4.07543E-05	0.007120485
TBC1D27P	-5.91332356	0.000623239	0.048028351
TLR8	-5.915457037	8.9161E-05	0.012754485
CLEC4A	-5.929209385	3.40066E-05	0.006413621
GNGT2	-5.944943126	0.000513106	0.042705366
TRAC	-6.053484034	9.60893E-05	0.013302342
LCP2	-6.059091235	8.4506E-06	0.002249039

AC078864.2	-6.062719866	1.66431E-05	0.003809273
CD86	-6.071437704	0.000100372	0.013674481
UNC5C	-6.125107797	2.06628E-05	0.004358571
MMP12	-6.157392909	8.64763E-05	0.012448236
OSM	-6.168962505	6.03542E-05	0.009562642
ITM2A	-6.199481208	7.67223E-06	0.002167926
ANGPT2	-6.249284391	1.06001E-09	1.73297E-06
POMC	-6.251510647	0.000569661	0.045272237
GIMAP5	-6.253652915	1.13305E-05	0.002841385
GIMAP6	-6.254589687	1.15689E-08	1.15125E-05
NXPE4	-6.336847465	0.000374934	0.03418924
CXCR4	-6.351328738	4.30809E-06	0.001350733
CCL4L2	-6.353410632	0.000247952	0.026427309
CDH5	-6.360711443	2.90065E-07	0.000175932
CD2	-6.376640591	3.59532E-05	0.006530929
GZMA	-6.379439029	7.20303E-06	0.002060786
SPIB	-6.391379934	8.36369E-06	0.002249039
ABI3	-6.406880794	2.82847E-07	0.000175932
F2RL2	-6.413440085	3.48515E-05	0.006415567
NKG7	-6.454406123	8.25779E-05	0.012193821
GPR183	-6.456674357	4.27586E-06	0.001350733
CD28	-6.493304645	0.000192891	0.021748187
HLA-DRA	-6.554070255	6.65503E-06	0.001952825
CD200	-6.582112706	0.000250837	0.026546662
RAMP3	-6.584612823	1.09983E-08	1.14423E-05
EXOC3L2	-6.63564095	1.82579E-09	2.61179E-06
NCF1C	-6.669581879	1.06724E-06	0.000469748
COMP	-6.682541455	8.47088E-05	0.012371613
PLXDC2	-6.688812611	8.3506E-07	0.000382257
GNLY	-6.711964876	1.2666E-05	0.003084038
ADGRF5	-6.731773041	6.84848E-11	2.60805E-07
CCL5	-6.732083629	4.07178E-05	0.007120485
CXCL9	-6.762640385	2.19612E-05	0.004569523
TRPC6	-6.773971984	4.90654E-05	0.008079194
PECAM1	-6.77518269	3.1976E-08	2.81487E-05
FHL5	-6.780534378	0.000493692	0.041542766
CCL4	-6.783600159	1.52732E-06	0.000624239
TCF15	-6.826137511	0.000197632	0.022065335
SFMBT2	-6.889123916	3.31609E-05	0.006324892
GIMAP8	-6.958600146	1.73745E-06	0.000685635
FLT4	-6.979611063	4.78829E-08	3.78181E-05
GJA5	-6.986254733	6.88724E-05	0.010723477
FOXS1	-6.995400636	4.55504E-05	0.007665857
AC087292.1	-7.003855639	0.000321232	0.030875307
IGHV3-66	-7.004066649	0.00035622	0.033008719
RGS18	-7.026903672	0.000120862	0.015990155
CD80	-7.027594188	8.62364E-05	0.012448236
TRBV20-1	-7.063502483	0.000118823	0.015904186
DCLK3	-7.083170856	0.000257467	0.02668725
ACAN	-7.098479602	8.70606E-07	0.000390714
RGS1	-7.126565053	2.06426E-10	5.24964E-07
ADGRL4	-7.158221431	6.4814E-09	7.8077E-06
AVPR1A	-7.218662898	0.000191667	0.021748187
GJA4	-7.259850473	5.69054E-08	4.3415E-05
IGKV1D-39	-7.299110516	0.000320521	0.030875307
CBFA2T3	-7.31145015	8.61574E-06	0.002249744
TLR7	-7.332411611	3.86832E-05	0.006917033
SFTPB	-7.340988027	2.83349E-07	0.000175932
CDH19	-7.365198526	0.000477383	0.040758495
CYYR1	-7.38156511	6.77571E-08	5.00266E-05
IGHG2	-7.391863263	5.98061E-07	0.000318335
CD34	-7.398126387	3.51486E-14	4.02241E-10
LINC01679	-7.411540063	0.000255023	0.02668725
EGFLAM	-7.415083404	2.30857E-06	0.000880643

IGHGP	-7.452948104	0.000459078	0.039952025
LINGO1	-7.457310612	0.000454654	0.039718046
PCDH17	-7.462844634	2.83952E-06	0.001015482
IGHV1-46	-7.483799575	1.80624E-05	0.004052188
ANOS1	-7.494431321	4.22681E-06	0.001350733
ICOS	-7.501229679	0.000195709	0.021957782
NPR1	-7.573412405	2.01754E-07	0.000139932
MS4A1	-7.580598731	1.3274E-05	0.003198055
CD93	-7.62491528	1.69295E-08	1.61451E-05
GZMK	-7.654289047	5.80082E-06	0.001724276
IGKV2D-28	-7.738281632	3.18597E-05	0.006219718
AC007192.2	-7.805812886	5.51765E-06	0.001683839
AC093278.2	-7.830252418	1.19445E-05	0.002939642
CLEC14A	-7.851355343	1.46271E-10	4.18481E-07
KDR	-7.865057414	4.08482E-11	1.86987E-07
SH2D1A	-7.895276704	7.38462E-05	0.011267941
SOX18	-7.936219073	7.97639E-11	2.60805E-07
CD79B	-7.962185362	7.18593E-07	0.000349939
PLVAP	-8.027666426	1.93496E-18	4.42874E-14
GIMAP4	-8.098554714	1.54952E-09	2.36436E-06
GZMH	-8.141300811	0.000212963	0.023322034
AC090984.1	-8.176118759	1.46892E-06	0.000615047
CLEC5A	-8.23571112	7.86653E-05	0.011767917
CCL17	-8.280721122	0.000426313	0.037819557
HRH2	-8.284711562	0.000126108	0.016307086
MEOX1	-8.306093861	1.82356E-05	0.004052188
COL22A1	-8.327569886	1.36603E-05	0.003256832
CLEC10A	-8.334675244	0.000159781	0.019247673
ROS1	-8.358499319	0.000519628	0.042935904
FDCSP	-8.366641644	0.000206537	0.022774175
NCF1B	-8.436411216	3.63966E-05	0.006559419
KMO	-8.440065566	5.66855E-05	0.009136741
SOX17	-8.477689553	5.50706E-07	0.000307428
AC020656.1	-8.479038895	0.000544936	0.044197835
BHLHE22	-8.678882804	4.7917E-08	3.78181E-05
CXCL13	-8.689948225	0.000206966	0.022774175
P2RY14	-8.892389916	2.81346E-06	0.001015482
MYCT1	-9.094356225	2.49548E-06	0.000936338
CALHM6	-9.135598091	5.32866E-07	0.000304906
ECSCR	-9.371007603	2.41825E-08	2.21396E-05
AC012615.2	-9.829593704	8.3859E-06	0.002249039
APLNR	-11.12484953	7.07028E-09	8.09123E-06
AC008050.1	-16.1834479	0.000305476	0.030398887
AL356488.2	-20.43841665	0.000280553	0.028287671
AC124068.1	-21.23197625	2.92092E-07	0.000175932
AC125611.3	-22.52951624	1.47796E-06	0.000615047

Table S4. Significantly enriched GO terms in organoid forming tumors compared to organoids.

Annotated Functions	*p*-Value	Number of Genes
immune response	1,3E-13	33
inflammatory response	1,3E-6	23
cell surface receptor signaling pathway	2,4E-5	18
signal transduction	3,2E-5	38
transmembrane receptor protein tyrosine kinase signaling pathway	7,7E-5	11
adaptive immune response	5,0E-4	12
superoxide metabolic process	6,5E-4	6
cell adhesion	7,4E-4	20
T cell costimulation	7,5E-4	9
positive regulation of ERK1 and ERK2 cascade	1,5E-3	12
regulation of immune response	1,6E-3	12

respiratory burst	1,5E-3	5
chemokine-mediated signaling pathway	2,6E-3	8
endothelium development	3,2E-3	4
positive regulation of cytosolic calcium ion concentration	3,7E-3	10
B cell receptor signaling pathway	4,1E-3	7
innate immune response	7,0E-3	17
cellular response to cytokine stimulus	9,5E-3	5
chemotaxis	9,1E-3	9
neutrophil chemotaxis	1,0E-2	7
extracellular matrix organization	1,0E-2	11
positive regulation of GTPase activity	1,4E-2	19
T cell activation	1,5E-2	6
cell-cell signaling	1,7E-2	12
G-protein coupled receptor signaling pathway	1,7E-2	25
microglial cell activation	1,8E-2	4
positive regulation of interleukin-2 biosynthetic process	1,8E-2	4
angiogenesis	2,2E-2	11
cellular response to interferon-gamma	3,0E-2	6
respiratory gaseous exchange	3,3E-2	5
leukocyte migration	3,3E-2	8
Fc-gamma receptor signaling pathway involved in phagocytosis	4,0E-2	8
antigen processing and presentation of peptide or polysaccharide antigen via MHC class II	4,2E-2	4
dendritic cell chemotaxis	4,2E-2	4
regulation of blood pressure	4,6E-2	6

© 2020 by the authors. Licensee MDPI, Basel, Switzerland. This article is an open access article distributed under the terms and conditions of the Creative Commons Attribution (CC BY) license (http://creativecommons.org/licenses/by/4.0/).
